# Application of novel Fe_3_O_4_/Zn-metal organic framework magnetic nanostructures as an antimicrobial agent and magnetic nanocatalyst in the synthesis of heterocyclic compounds

**DOI:** 10.3389/fchem.2022.1014731

**Published:** 2022-10-10

**Authors:** Bashar S. Bashar, Hawraa A. Kareem, Yaser Mohamed Hasan, Nafis Ahmad, A. M. Alshehri, Kadhum Al-Majdi, Salema K. Hadrawi, Munthir Mohammed Radhy AL Kubaisy, Maytham T. Qasim

**Affiliations:** ^1^ Department of Computing Technologies Engineering, Al-Nisour University College, Baghdad, Iraq; ^2^ Anesthesia Techniques Department, Al-Mustaqbal University College, Babylon, Iraq; ^3^ Technical Engineering College, Al-Farahidi University, Baghdad, Iraq; ^4^ Department of Physics, College of Science, King Khalid University, Abha, Saudi Arabia; ^5^ Department of Biomedical Engineering, Ashur University College, Baghdad, Iraq; ^6^ Refrigeration and Air-conditioning Technical Engineering Department, College of Technical Engineering, The Islamic University, Najaf, Iraq; ^7^ The University of Mashreq, Baghdad, Iraq; ^8^ Department of Anesthesia, College of Health and Medical Technology, Al-Ayen University, Thi-Qar, Iraq

**Keywords:** Fe3O4/Zn-metal organic framework magnetic nanostructures, microwave assisted, antimicrobial agent, MIC and MBC value, spiro[indoline-pyranopyrimidines

## Abstract

Using the microwave-assisted method, novel Fe_3_O_4_/Zn-metal organic framework magnetic nanostructures were synthesized. The crystallinity, thermal stability, adsorption/desorption isotherms, morphology/size distribution, and magnetic hysteresis of synthesized Fe_3_O_4_/Zn-metal organic framework magnetic nanostructures were characterized by XRD patterns, TGA curve, BET adsorption/desorption technique, SEM image, and VSM curve, respectively. After confirming the Fe_3_O_4_/Zn-metal organic framework magnetic nanostructures, its antimicrobial properties against Gram-positive bacterial, Gram-negative bacterial, and fungal strains based on minimum inhibitory concentration (MIC), minimum bactericidal concentration (MBC), and minimum fungicidal concentration (MFC) values were studied. The MIC values in antimicrobial activity for Gram-positive and Gram-negative bacterial strains, between 16–128 μg/ml, and for fungal strain, 128 μg/ml were observed. The results showed that the high specific surface area of Fe_3_O_4_/Zn-metal organic framework magnetic nanostructures caused the antimicrobial power of nanoparticles to be high, and the observed antimicrobial effects were higher than some known commercial antimicrobial drugs. Another advantage of the specific surface area of Fe_3_O_4_/Zn-metal organic framework magnetic nanostructures was its high catalytic properties in the three-component reaction of isatin, malononitrile, and dimedone. New spiro [indoline-pyranopyrimidines] derivatives were synthesized with high efficiency. The catalytic activity results of Fe_3_O_4_/Zn-metal organic framework magnetic nanostructures showed that, in addition to recyclability, derivatives could be synthesized in less time than previously reported methods. The results of investigating the catalytic activity of Fe_3_O_4_/Zn-metal organic framework magnetic nanostructures showed that the spiro [indoline-pyranopyrimidines] derivatives were synthesized in the time range of 10–20 min with an efficiency of over 85%. As a final result, it can be concluded that the microwave synthesis method improves the unique properties of magnetic nanostructures, especially its specific surface area, and has increased its efficiency.

## 1 Introduction

Organometallic framework compounds with crystalline structures and unique properties have attracted the attention of chemists. These efficient nanostructures have been synthesized by different methods, and various applications have been reported for them (Ren et al., 2015; Al-Rowaili et al., 2018). These nanostructures have excellent physical and chemical properties, based on these properties; they show broad and diverse applications. High stability against heat, high porosity, high resistance on the surface, and high reactivity was the capabilities of these compounds (Ghanbari et al., 2020). Recently, the use of MOF nanostructures as a catalyst in synthesizing organic compounds, especially heterocycles and their derivatives, has been expanding (Pascanu et al., 2019; Dhameliya et al., 2020; Goetjen et al., 2020; Purohit et al., 2020). MOF compounds with magnetic properties have also been reported so far. The importance of these compounds in the synthesis of organic compounds is their easy separation after performing the reaction by a magnet (Yao et al., 2016; Ghorbani-Choghamarani and Taherinia, 2020). Biological properties such as enzyme immobilization, enantioselective hydrolysis of (R, S)-naproxen methyl ester, and immobilization of proline activated lipase from magnetic MOF compounds have been reports (Nadar and Rathod, 2018; Nadar and Rathod, 2020; Ozyilmaz et al., 2021).

In the synthesizing of organic compounds, spiro heterocycles play a unique role, and the synthesis of these compounds has been widely reported in modern chemistry (Fatahpour et al., 2017; Pogosyan et al., 2018). Investigations show that spiroheterocycle compounds have biological properties such as anticancer, antianaphylactic, anticoagulant, spasmolytic activities, and the synthesis procedure of these compounds has been considered due to these properties (Chen et al., 1999; Saraswat et al., 2016; Harichandran et al., 2018; Faroughi Niya et al., 2021; Al-Obaidi et al., 2022).

Pyrimidines are the main building blocks of nucleic acid. In addition, a literature review shows that pyrimidines have many biological properties such as anti-HIV agents, antitumor activity, anti-inflammatory activity, antimalarial activity, anti-microbial activity, antihypertensive activity, potassium channel antagonists, etc. (Bhat, 2017; Ahmad et al., 2021; Ahmad et al., 2022).

Pyran derivatives also have many biological properties. Biological properties such as anticancer (Sayed et al., 2019), antiviral activity (Morahan et al., 1972; Shehab and Ghoneim, 2016), antibacterial (Shehab and Ghoneim, 2016; Dutta et al., 2021), *etc*. Have also been reported from heterocyclic compounds containing pyran derivatives.

Pyranopyrimidines are one of the essential polycyclic heterocyclic compounds with high biological properties. Biological properties such as antimicrobial activities (Bedair et al., 2001), anti-HIV activity (Maddila et al., 2020), antioxidant activity (Yousefi et al., 2015), anticancer activity (Bhosle et al., 2019), and antitumor activity (Haggam et al., 2020) from polycyclic heterocyclic compounds contain pyranopyrimidines derivatives have been reported.

Multicomponent reactions are an essential method in synthesizing of heterocyclic, and organic compounds and many ways have been reported in this regard (Biswas and Das, 2022; Coppola et al., 2022; Wang et al., 2022). One of the crucial factors in multicomponent reactions is the selection of the appropriate catalyst. Several catalysts such as nanoparticles and magnetic nanoparticles have been reported to synthesize organic and heterocyclic compounds in multicomponent reactions (Amoozadeh et al., 2016; Chen et al., 2019; Ghorbani-Choghamarani et al., 2019; Harikrishna et al., 2020; Arlan et al., 2021).

Considering the importance of the synthesis of new nano compounds with high capabilities, in this research, novel Fe_3_O_4_/Zn-metal organic framework magnetic nanostructures were synthesized. After confirming their structure and determination of antimicrobial properties, they were used as magnetic catalysts in the three-component synthesis of new spiro [indoline-pyranopyrimidines] derivatives. Significant results of nanoparticles in antimicrobial and catalytic properties were observed.

## 2 Experimental

### 2.1 General

**FIGURE 1:**
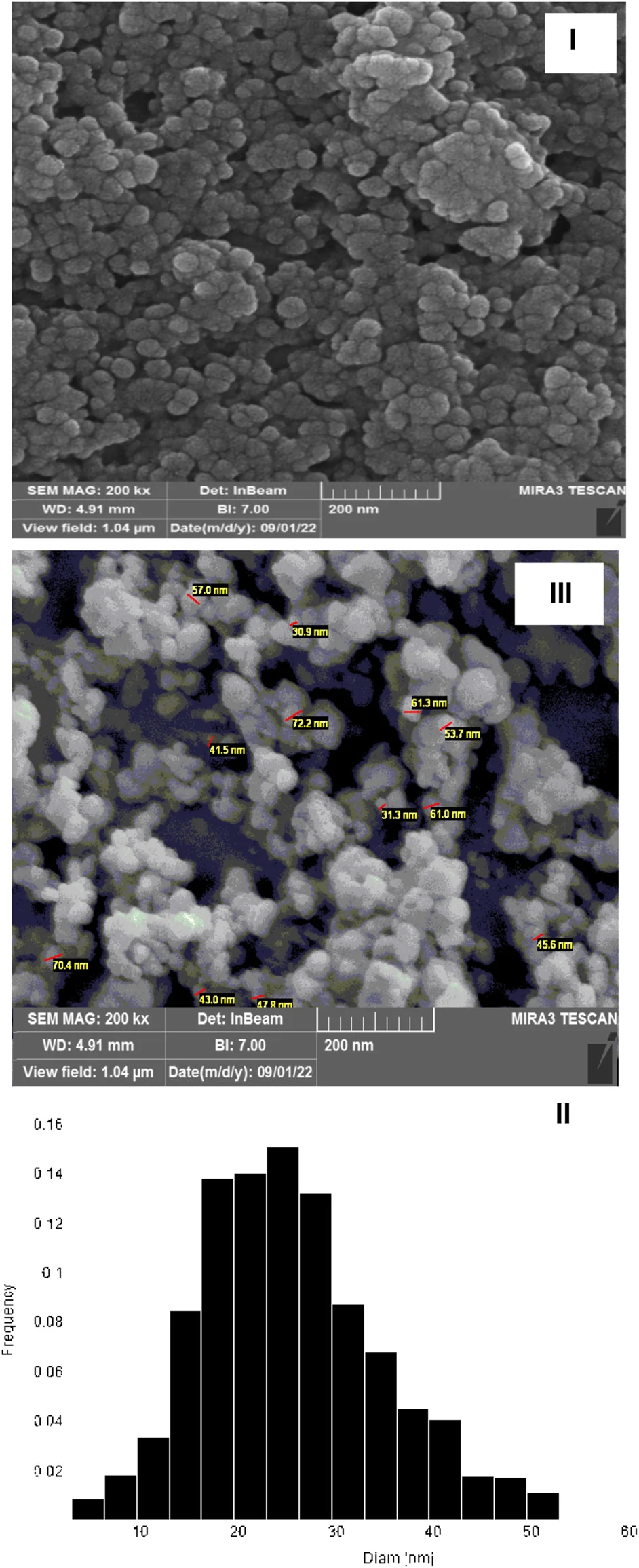
SEM image (I) and size histogram (II) of Fe_3_O_4_/Zn-metal organic framework magnetic nanostructures and SEM image of Fe_3_O_4_ (III).

**FIGURE 2:**
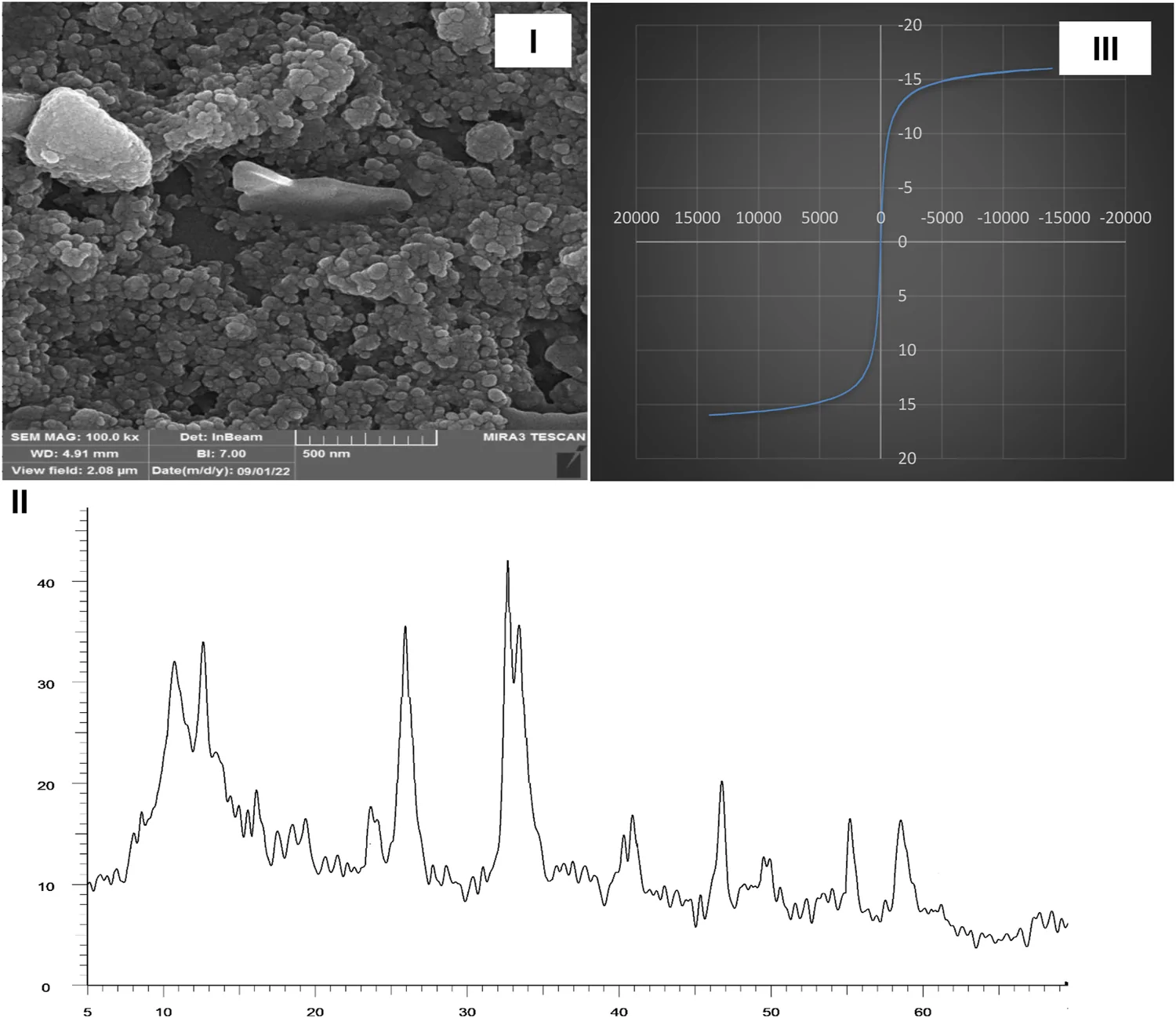
SEM (I), XRD (II), and VSM (III) of Fe_3_O_4_/Zn-metal organic framework magnetic nanostructures reusability in the synthesis of compound 4a.

The SEM images were obtained using a TESCAN MIRA3 field-emission scanning electron microscope (FE-SEM). The micrographs presented in Figures 1 (I, III) and 2 (I) were acquired at an accelerating voltage of 15.0 kV and a working distance of 4.91 mm using an InBeam detector. Figures 1 (I, III) were recorded at a magnification of 200 k×, whereas Figure 2 (I) was recorded at a magnification of 100 k×. Information regarding conductive coating was not documented in the retained experimental records. The FT-IR spectra were recorded by Nicolet AVATAR 360 FT-IR spectrophotometer. An advanced microwave synthesis lab station (MICROSYNTH, Milestone Co.) was used to synthesize Fe_3_O_4_/Zn-metal organic framework magnetic nanostructures. By Bruker FT-NMR Ultra Shield-spectrometer (300 and 75 MHz), the ^1^H- and ^13^C-NMR spectra were recorded. Uncorrected melting points of derivatives by KSP1N melting point meter of Krus’s type were determined.

### 2.2 Synthesis of Fe_3_O_4_/Zn-metal organic framework magnetic nanostructures by using microwave irradiation

For the synthesis of Fe_3_O_4_/Zn-metal organic framework magnetic nanostructures by microwave method, ZnCl_2_ (2°mmol), pyridine-2,6-dicarboxylic acid (4°mmol), and Fe_3_O_4_ nanoparticle (1°mmol), were added to the mixture including double-distilled water/acetic acid (30°ml, 1:1) and stirred quickly 15°min at 70 °C. In the next step, the mixture was subjected to microwave irradiation with a power of 350°W for 20°min at room temperature. Finally, by using a magnet, the Fe_3_O_4_/Zn-metal organic framework magnetic nanostructures were separated and washed several times with water and acetic acid and dried under vacuum at ambient temperature (Sargazi et al., 2018).

### 2.3 Antimicrobial activity of Fe_3_O_4_/Zn-metal organic framework magnetic nanostructures

To obtain the antimicrobial property of Fe_3_O_4_/Zn-metal organic framework magnetic nanostructures based on MIC, MBC, and MFC on Gram-negative, Gram-positive, and fungal strains, the clinical and laboratory standards institute (CLSI) guidelines M07-A9, M26-A, M02-A11, M44-A, and M27-A2, and previously reported methods were used (Moghaddam-Manesh et al., 2020; Abdieva et al., 2022; Zeraati et al., 2022).

### 2.4 Synthesis of 1-benzylindoline-2,3-dione

By using indoline-2,3-dione, benzyl halide, potassium carbonate, and potassium iodide in acetonitrile and the method reported by Auria-Luna et al. Auria-Luna et al. (2015) and Tehrani et al. Tehrani et al. (2016) 1-benzylindoline-2,3-dione (3f) was synthesized. 1-Benzylindoline-2,3-dione was used as a reactant to synthesize new spiro [Indoline-pyranopyrimidine] derivatives.

### 2.5 General procedure for the synthesis of spiro [Indoline-pyranopyrimidine] derivatives

For the synthesis of spiro [indoline-pyranopyrimidines] derivatives, malononitrile (1 mmol), indoline-2,3-dione derivatives (1 mmol), barbituric acid or thiobarbituric acid (1 mmol), and 0.03 g catalyst (Fe_3_O_4_/Zn-metal organic framework magnetic nanostructures) added to 2 ml EtOH. The resultant was stirred at room temperature (optimal condition). The progress of the reaction was monitored by thin layer chromatography (TLC). After completion of the reaction, the catalyst was separated using a magnet. Finally, recrystallization of the mixture in water and ethanol was used to purify the sediments.

#### 2.5.1 7′-amino-1-benzyl-2,2′,4′-trioxo-1′,2′,3′,4′-tetrahydrospiro [indoline-3,5′-pyrano [2,3-d]pyrimidine]-6′-carbonitrile (4K)

IR (KBr, ν, cm^−1^): 3290, 3350 (NH_2_), 3156 (NH), 2940 (CH), 2193 (CN), 1726 (CO), 1487 (C=C).

^1^H NMR (300 MHz, DMSO-*d*_6_): δ 4.25 (s, 2H, CH_2_), 6.73 (d, 1H, *J* = 8.4 Hz, ArH), 6.88–6.89 (t, 1H, ArH), 7.01–7.12 (m, 4H, ArH), 7.24 (d, 2H, *J* = 8.4 Hz, ArH), 7.37–7.8 (t, 1H, ArH), 7.45 (s, 2H, NH_2_), 10.64 (s, 1H, NH), 12.41 (s, 1H, NH) ppm.

^13^C NMR (75 MHz, DMSO-*d*_6_): *δ* 184.17, 163.29, 158.34, 154.07, 149.73, 140.86, 136.12, 134.57, 129.36, 128.75, 128.42, 127.46, 127. 55, 126.48, 124.55, 122.07, 117.45, 109.18, 88.52, 58.24, 55.01, 47.76 ppm.

Elemental analysis (C_22_H_15_N_5_O_3_S): Calculated; C, 61.53; H, 3.52; N, 16.31; S, 7.47. Found: C, 61.57; H, 3.49; N, 16.32; S, 7.50.

#### 2.5.2 7′-amino-1-benzyl-2,4′-dioxo-2′-thioxo-1′,2′,3′,4′-tetrahydrospiro [indoline-3,5′-pyrano [2,3-d]pyrimidine]-6′-carbonitrile (4L)

IR (KBr, ν, cm^−1^): 3420, 3364 (NH_2_), 3171 (NH), 2958 (CH), 2195 (CN), 1717 (CO), 1522 (C=C).

^1^H NMR (300 MHz, DMSO-*d*_6_): δ 4.32 (s, 2H, CH_2_), 6.77 (d, 1H, *J* = 8.7 Hz, ArH), 6.84–6.91 (t, 1H, ArH), 7.04–7.10 (m, 4H, ArH), 7.21 (d, 2H, *J* = 8.4 Hz, ArH), 7.30–7.37 (t, 1H, *J* = 8 Hz, ArH), 7.42 (s, 2H, NH_2_), 11.04 (s, 1H, NH), 12.01 (s, 1H, NH) ppm.

^13^C NMR (75 MHz, DMSO-*d*_6_): *δ* 172.38, 162.45, 158.02, 154.17, 149.28, 141.01, 135.74, 134.28, 129.37, 128.64, 128.31, 127.31, 127. 01, 126.29, 124.29, 122.76, 117.69, 109.47, 88.31, 58.44, 54.67, 47.23 ppm.

Elemental analysis (C_22_H_15_N_5_O_4_): Calculated; C, 63.92; H, 3.66; N, 16.94; O, 15.48. Found: C, 63.90; H, 3.69; N, 16.95, O, 15.46.

## 3 Results and discussion

### 3.1 Characterization of Fe_3_O_4_/Zn-metal organic framework magnetic nanostructures

**FIGURE 3:**
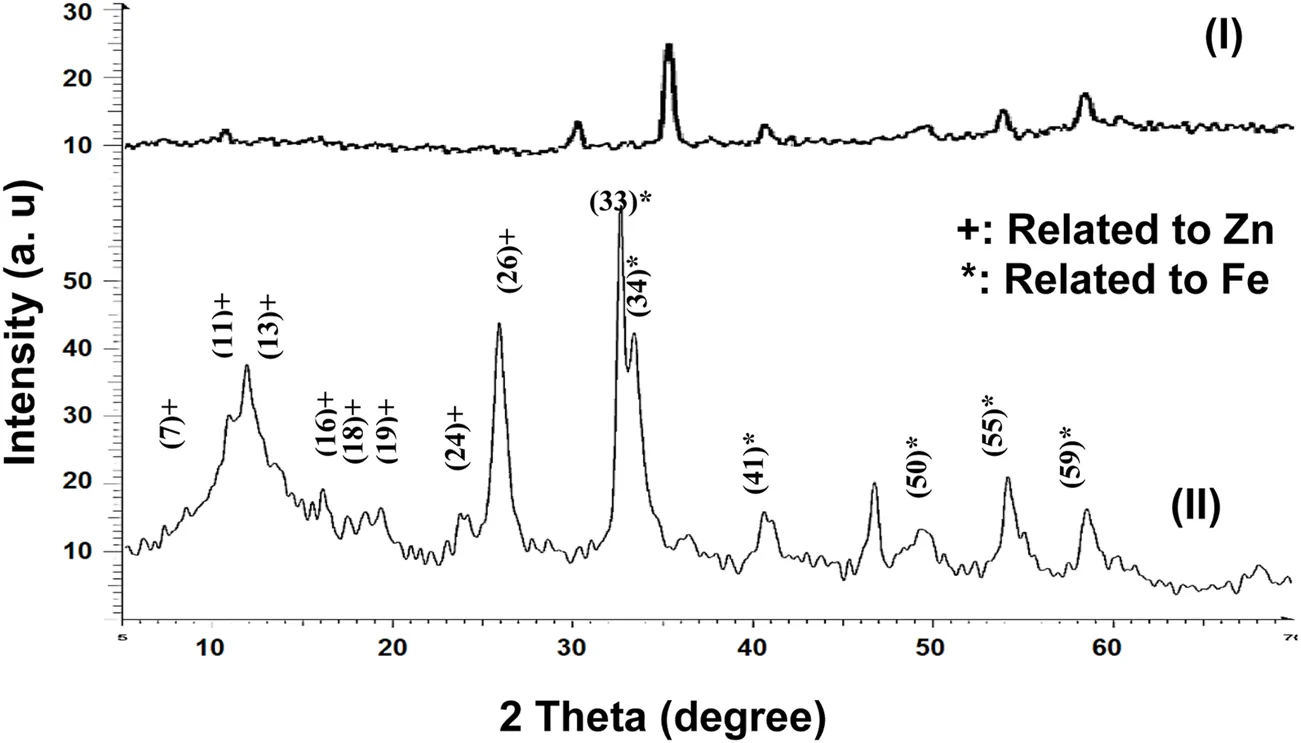
XRD patterns of Fe_3_O_4_ (I) Fe_3_O_4_/Zn-metal organic framework magnetic nanostructures (II).

The pattern given in Figure 3 showed the XRD pattern of Fe_3_O_4_/Zn-metal organic framework magnetic nanostructures. The obtained XRD pattern was similar to the standard pattern reported for zinc and Fe_3_O_4_ nanoparticles (Hosseinzadegan et al., 2020; Rafiee, 2021; Zeraati et al., 2022). The calculation of Debby Scherer’s equation for Fe_3_O_4_/Zn-metal organic framework magnetic nanostructures showed that the size of the synthesized nanoparticles is 25 nm, which proves the importance of the synthesis method using microwaves.

**FIGURE 4:**
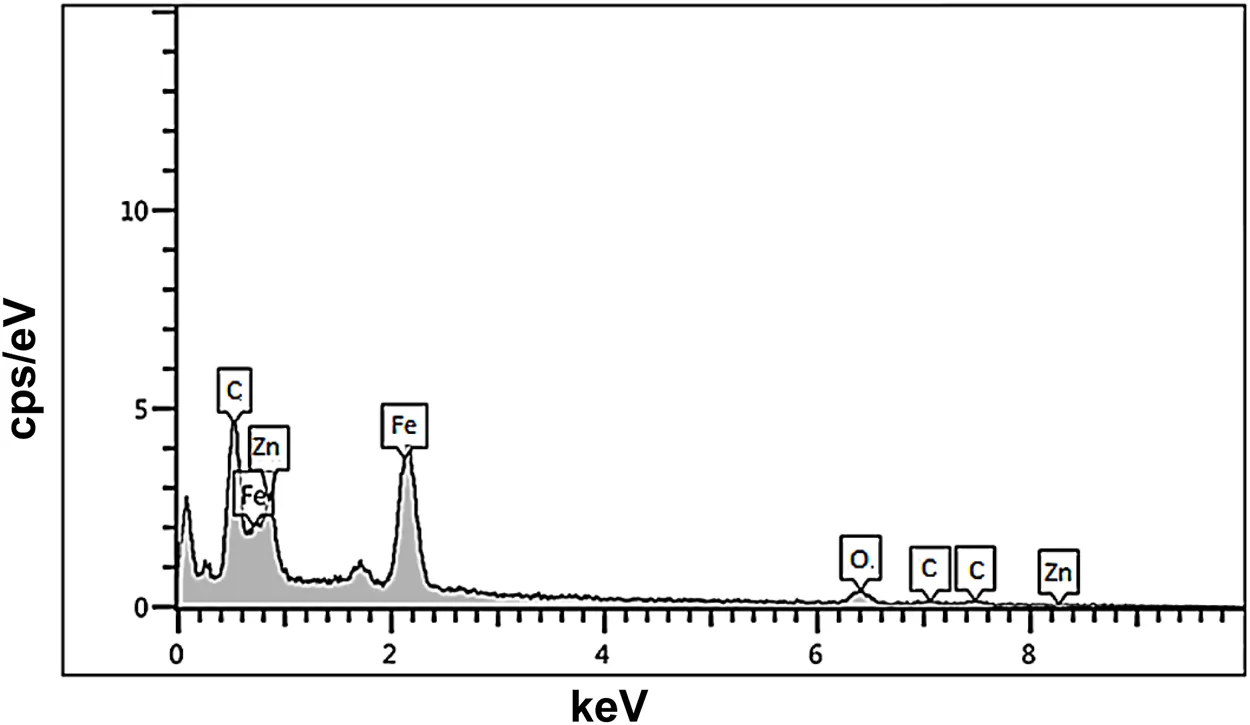
EDX spectrum of Fe_3_O_4_/Zn-metal organic framework magnetic nanostructures.

In Figure 4, EDX spectrum of Fe_3_O_4_/Zn-metal organic framework magnetic nanostructures were given which confirmed the successful synthesis of products.

Based on the EDX spectrum of Fe_3_O_4_/Zn-metal organic framework magnetic nanostructures, the elements in the raw materials, including C, Fe, Zn, and O, were observed in the final product.

**FIGURE 5:**
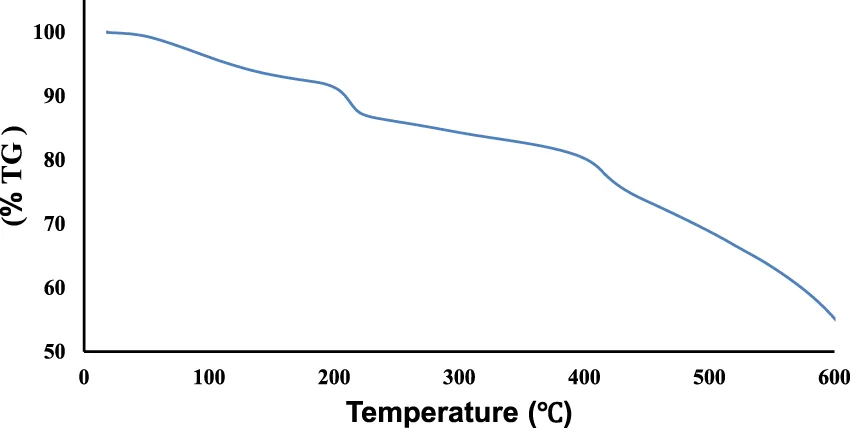
Thermal stability curve of Fe_3_O_4_/Zn-metal organic framework magnetic nanostructures.

The results obtained from the thermal stability curve of Fe_3_O_4_/Zn-metal organic framework magnetic nanostructures show that in the temperature range of 90–100 °C, the partial weight decreases due to the evaporation of the solvent on the surface of the sample (Figure 5). The partial weight loss in the second stage in the temperature range of about 190 C is related to the evaporation of the solvent trapped in the nanoparticle structure. In the third stage, a noticeable weight loss in the sample was observed first in the range of 392 C of the pure Fe_3_O_4_/Zn-metal organic framework magnetic nanostructures. Then, at near 600 C, we see the beginning of the degradation and collapse of the final Fe_3_O_4_/Zn-metal organic framework magnetic nanostructures. From the observations, it can be concluded that Fe_3_O_4_/Zn-metal organic framework magnetic nanostructures had high thermal stability. The high thermal stability can be provided the advantages and potential applications of these synthesized compounds in the optimal microwave method.

**FIGURE 6:**
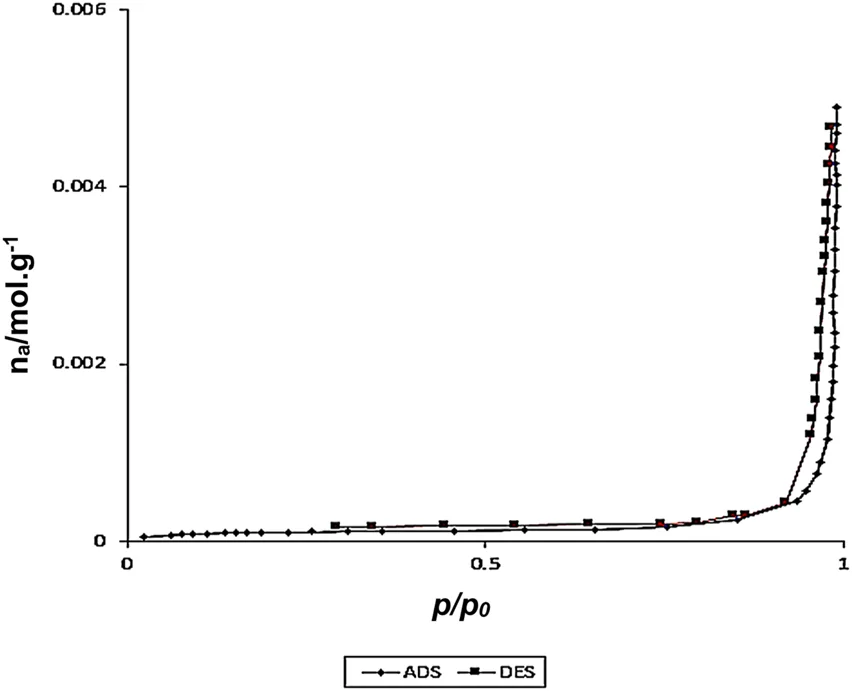
N_2_ adsorption/desorption isotherm of Fe_3_O_4_/Zn-metal organic framework magnetic nanostructures.

The following graphs (Figure 6) were obtained using N_2_ adsorption and desorption techniques from core-shell nanostructures of Fe_3_O_4_/Zn-metal organic framework magnetic nanostructures. The BET diagram of Fe_3_O_4_/Zn-metal organic framework magnetic nanostructures shows that the effective specific surface area was corresponded to the final core-shell nanostructure.

The specific surface area for the Fe_3_O_4_/Zn-metal organic framework magnetic nanostructures was about 37,500 m^2^/g. The high specific surface area is an essential factor in the effectiveness of nanoparticles in catalytic reactions and biological properties. It can be related that the use of microwave method in the synthesis of these structures was an important reason for the high specific surface as well as high thermal stability of Fe_3_O_4_/Zn-metal organic framework magnetic nanostructures.

**FIGURE 7:**
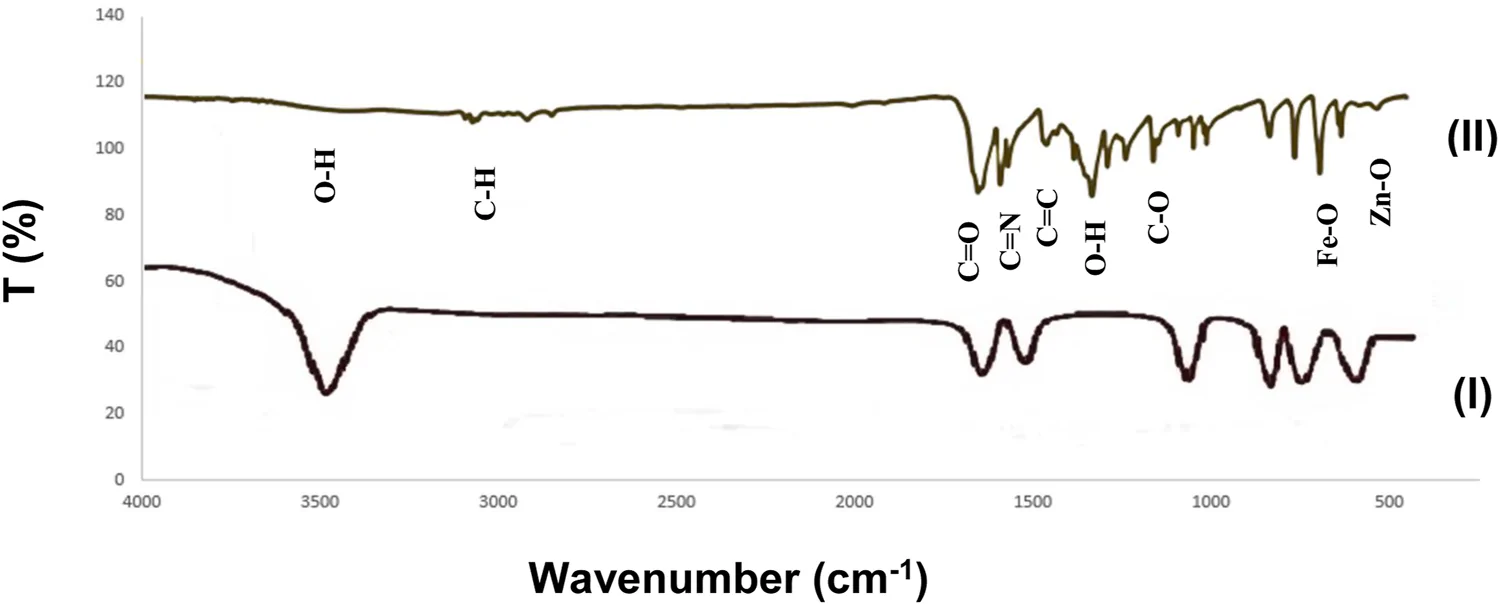
FTIR spectrum of Fe_3_O_4_ (I) and Fe_3_O_4_/Zn-metal organic framework magnetic nanostructures (II).

The figure below (Figure 7) shows the FTIR spectrum of Fe_3_O_4_/Zn-metal organic framework magnetic nanostructures synthesized by the microwave route. According to the obtained spectrum, the broad peak in the 3500 cm^−1^ was related to water molecules and OH groups coordinated to the Fe_3_O_4_/Zn-metal organic framework magnetic nanostructures. The absorption around the 3100 and 2900 cm^−1^ region is related to the C-H stretching bond in the aromatic ring. The frequency in the region of about 1653 cm^−1^ corresponds to the -COO- group present in the final structure of Fe_3_O_4_/Zn-metal organic framework magnetic nanostructures. The peaks in the region ∼1590 cm^−1^ were due to stretching bond of C=N, C=C stretching bond appears in 1450 cm^−1^, the peak in area 1335 cm^−1^ for O-H bending, C-O groups appear in 1162 cm^−1^. The peck in rejoins 630 cm^−1^ was related to Fe-O (Togashi et al., 2011), and finally, the peak in rejoin 500 cm^−1^ was attributed to Zn-O (Rafiee, 2021).

Figure 1 shows the SEM image and particle size histogram of Fe_3_O_4_/Zn-metal organic framework magnetic nanostructures. It seems that development of the microwave method with optimal conditions has led to the production of the MOF sample with uniform morphology and high stability surface. According to the SEM and size histogram, no effect of agglomeration of particles were observed, but nanoparticles were observed in a one-dimensional form (average particle size of 24 nm) with a clear correlation which can be attributed to the use of the efficient microwave synthesis method. The evidence shows that the type of synthesis method has a significant effect on the morphology and particle size distribution. As an important result, synthesis of nanostructures with uniform size distribution and homogeneous surfaces have special applications in medical science.

**FIGURE 8:**
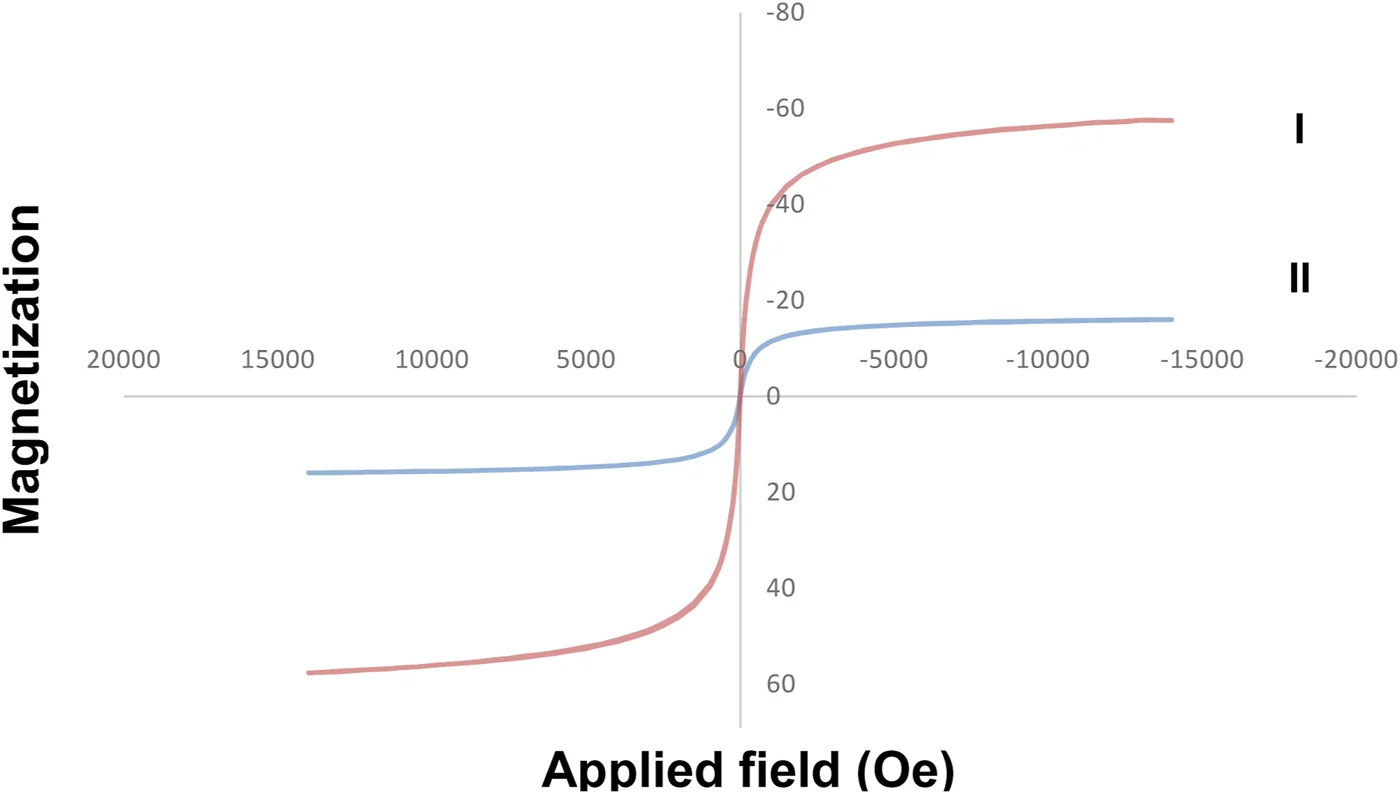
Magnetic property curve of Fe_3_O_4_ (I) and Fe_3_O_4_/Zn-metal organic framework magnetic nanostructures (II).

The figure below (Figure 8) shows the magnetic property of Fe_3_O_4_/Zn-metal organic framework magnetic nanostructures. The magnetic property for Fe_3_O_4_ MNPs 57 emu/g was reported (Shiri et al., 2018). The magnetic property of Fe_3_O_4_/Zn-metal organic framework magnetic nanostructures 16.1 emu/g was obtained and proved that the core (Fe_3_O_4_) were covered with Zn-metal organic framework magnetic nanostructures as a shell. The importance of magnetic properties in the synthesized nanostructures was revealed when they were separated from the reaction medium. The magnetic property makes the catalyst easily separated by a magnet after the reaction is finished.

**FIGURE 9:**
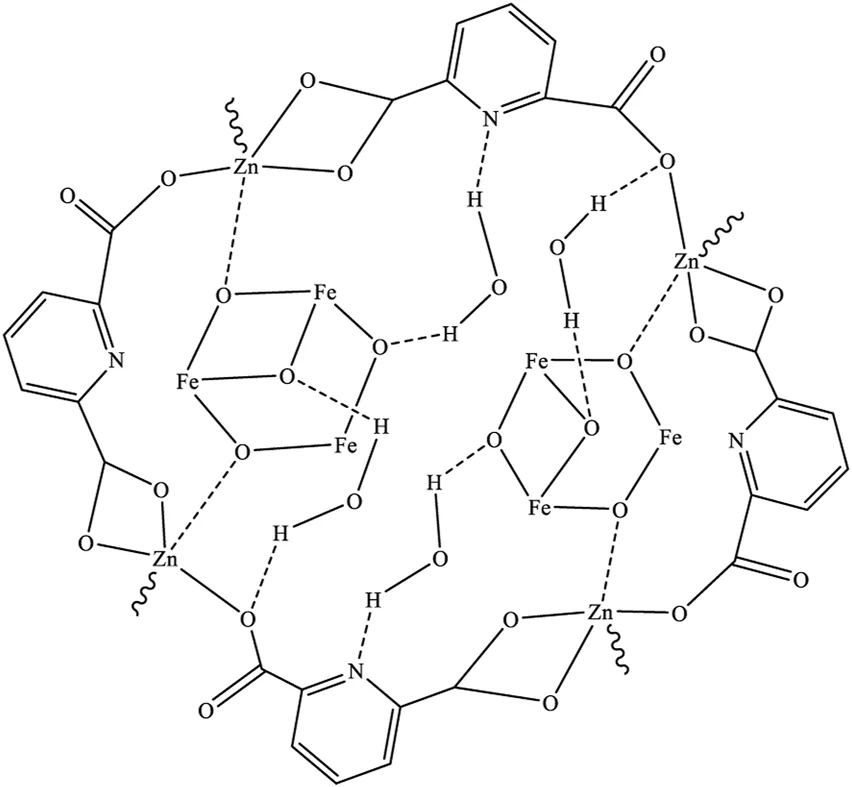
Proposed structure for Fe_3_O_4_/Zn-metal organic framework magnetic nanostructures.

Based on the obtained spectral data, the structure of Figure 9 was proposed for Fe_3_O_4_/Zn-metal organic framework magnetic nanostructures.

### 3.2 Antimicrobial activity of Fe_3_O_4_/Zn-metal organic framework magnetic nanostructures

In the investigation of antimicrobial activities, Gram-negative strains including, *Pseudomonas aeruginosa* and *Shigella dysenteriae*; Gram-positive bacteria strains including, *Rhodococcus equi* and *Streptococcus agalactiae*; Fungi including, *Candida albicans* were used.

Investigations showed that nanoparticles were effects on all Gram-positive and Gram-negative bacterial and fungal strains studied. The obtained results were given in Table 1.

**TABLE 1:** Antibacterial and Antifungal activity of Fe_3_O_4_/Zn-metal organic framework magnetic nanostructures.

Sample	Gram-negative bacteria				Gram-positive bacteria				Fungi	
	*Pseudomonas aeruginosa*		*Shigella dysenteriae*		*Rhodococcus equi*		*Streptococcus agalactiae*		*Candida albicans*	
	**MIC**	**MBC**	**MIC**	**MBC**	**MIC**	**MBC**	**MIC**	**MBC**	**MIC**	**MFC**
	**(μg/ml)**	**(μg/ml)**	**(μg/ml)**	**(μg/ml)**	**(μg/ml)**					
A	64	128	32	64	32	64	16	32	128	256
B	32	64	0.5	1	4	16	-	-	32	64
C	-	-	-	-	-	-	8	16	-	-

A: Fe_3_O_4_/Zn-metal organic framework magnetic nanostructures; B: gentamicin for bacteria, Terbinafine for Fungi; C: cefazolin for bacteria, Tolnaftate for Fungi.

Fe_3_O_4_/Zn-metal organic framework magnetic nanostructures were effective against all study bacterial and fungal strains, and MBC values of 32–256 μg/ml were obtained. The effectiveness of Fe_3_O_4_/Zn-metal organic framework magnetic nanostructures against Gram-positive strains was more than Gram-negative strains and fungi. The effectiveness of Fe_3_O_4_/Zn-metal organic framework magnetic nanostructures was compared with the efficacy of commercial drugs in the market. More effectiveness of nanoparticles compared to drugs was observed. In general, the efficacy of Fe_3_O_4_/Zn-metal organic framework magnetic nanostructures can be attributed to their high specific surface that engages with bacterial and fungal strains.

### 3.3 Synthesis of spiro [indoline-pyranopyrimidines] derivatives by Fe_3_O_4_/Zn-metal organic framework magnetic nanostructures

**SCHEME 1:**
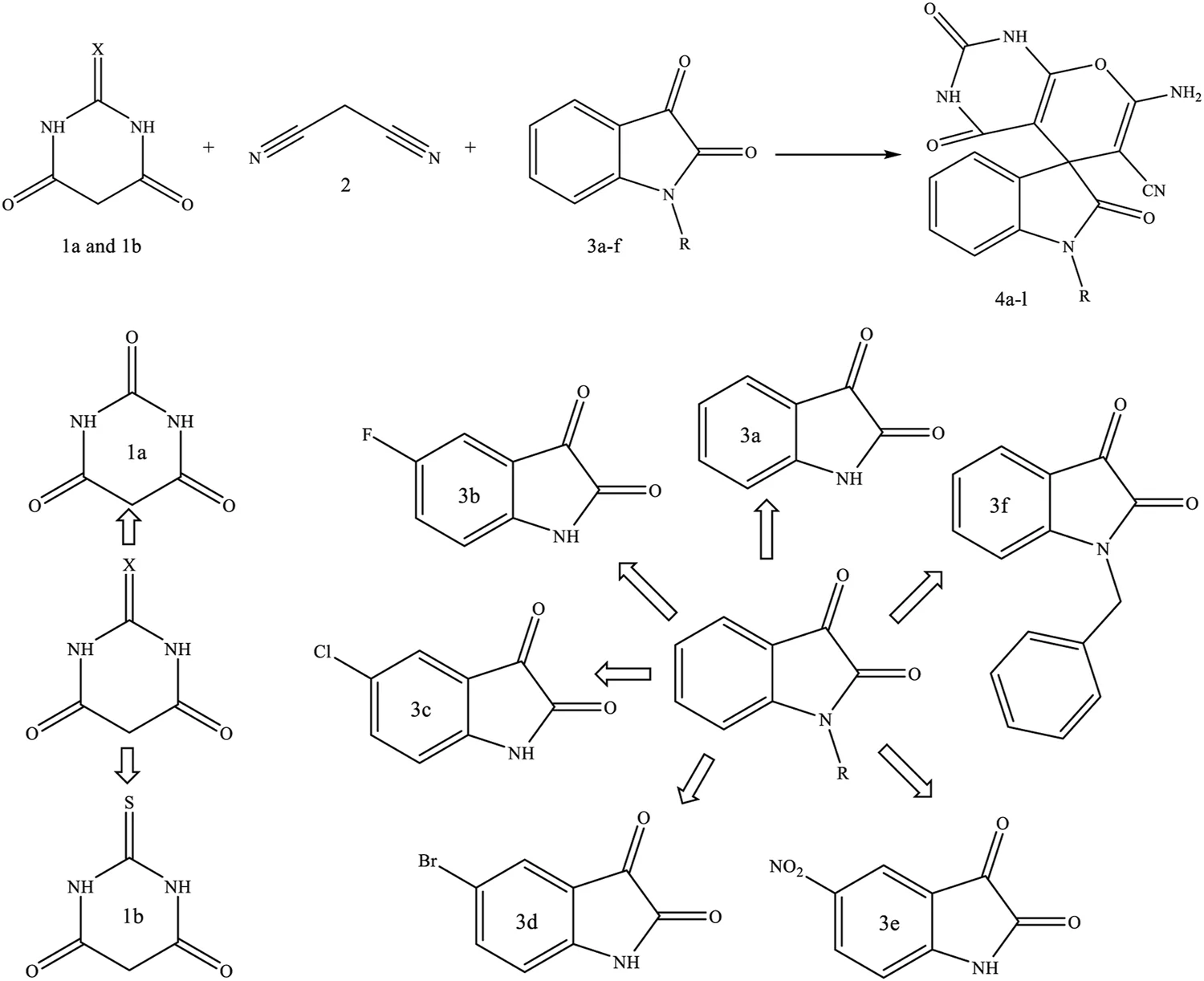
Fe_3_O_4_/Zn-metal organic framework magnetic nanostructures as magnetic nanocatalyst in synthesis spiro [indoline-pyranopyrimidines] derivatives.

In this study, based on Scheme 1, using Fe_3_O_4_/Zn-metal organic framework magnetic nanostructures as magnetic nanocatalyst during the three-component reaction of malononitrile, indoline-2,3-dione derivatives, and barbituric acid or thiobarbituric acid, new spiro [indoline-pyranopyrimidines] derivatives were synthesized.

**SCHEME 2:**
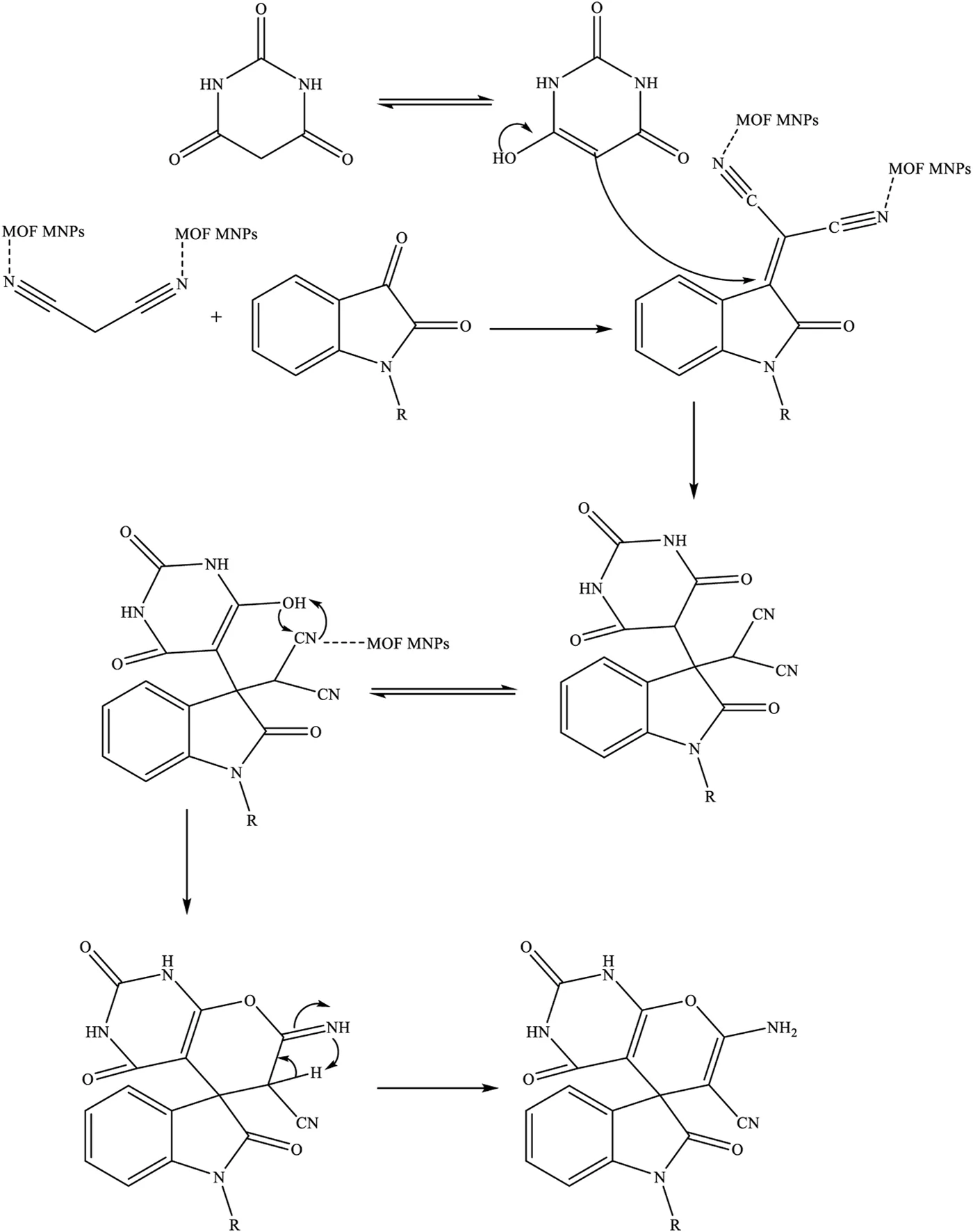
Proposed mechanisms for the synthesis of spiro [indoline-pyranopyrimidines] derivatives by Fe_3_O_4_/Zn-metal organic framework magnetic nanostructures

For the synthesis of spiro [indoline-pyranopyrimidines] derivatives, the optimal conditions of solvent, amount of catalyst, and temperature were studied according to Table 2 (for compound 4a).

**TABLE 2:** Determination of optimal conditions in synthesis of spiro [indoline-pyranopyrimidines] derivatives.

Product	Solvent	Catalyst (g)	Temperature (^o^C)	Time (min)	Yield (%)	TON	TOF (min^−1)^
4a	EtOH	0.01	r. t	30	81	45.76 × 105	152,500
4a	H_2_O	0.01	r. t	60	35	19.77 × 105	32,950
4a	H_2_O:EtOH (1:1)	0.01	r. t	30	72	40.68 × 105	135,600
4a	MeOH	0.01	r. t	60	64	36.16 × 105	60,270
4a	EtOH	0.02	r. t	30	89	25.21 × 105	84,030
4a	EtOH	0.03	r. t	10	97	18.30 × 105	183,000
4a	EtOH	0.04	r. t	10	94	13.30 × 105	133,000
4a	EtOH	0.05	r. t	10	88	9.97 × 105	99,700
4a	EtOH	0.03	40	10	90	16.98 × 105	169,800
4a	EtOH	0.03	50	10	83	15.66 × 105	156,600

The results of ICP showed that 0.03 g of Fe_3_O_4_/Zn-metal organic framework magnetic nanostructures contain ×3.4710^–3^ g of zinc or 5.3 × 10^–5^ mol or Zn, therefore in optimal conditions, TON and TOF were obtained, 18×10^5^ and 180,000 min^−1^, respectively.

According to Table 3, using optimal conditions studied in Table 1 (EtOH as a solvent, 0.03 mg of Fe_3_O_4_/Zn-metal organic framework magnetic nanostructures and room temperature), 12 spiro [indoline-pyranopyrimidines] derivatives were synthesized, and derivatives 4k and 4L were novel and reported for the first time.

**TABLE 3:** Synthesized derivatives of spiro [indoline-pyranopyrimidines] derivatives (4a-l) by Fe_3_O_4_/Zn-metal organic framework magnetic nanostructures.

Product	Structure	Time (min)	Yield (%)	TON	TOF (min^−1^)	M.p. (°C)	
						Found	Reported
	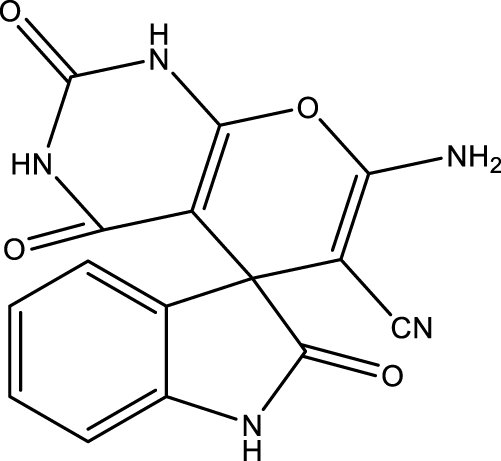	10	97	18.30 × 105	183,000	272–275	275 (Keshavarz and Farahi, 2022)
b	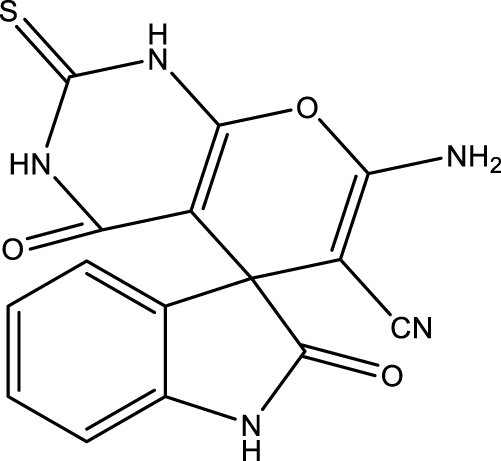	12	96	18.11 × 105	150,900	241–242	240–242 (Azimi and Lasemi, 2022)
4c	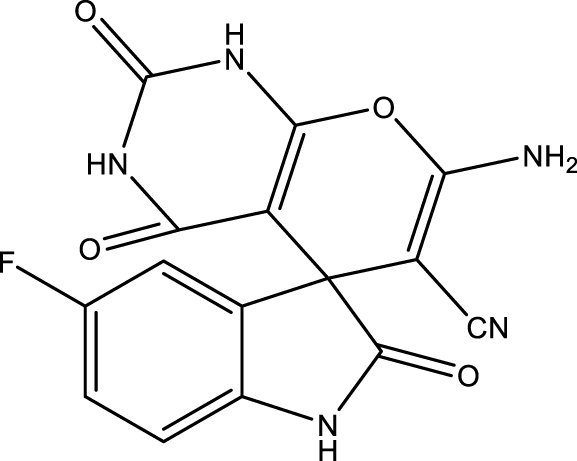	12	91	17.17 × 105	143,080	235–237	235 (Joshi et al., 1988)
4d	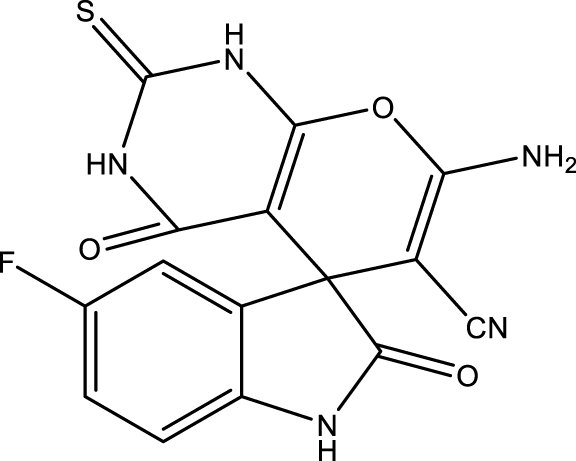	15	92	17.36 × 105	115,730	255–256	252–254 (Keshavarz, 2016)
4e	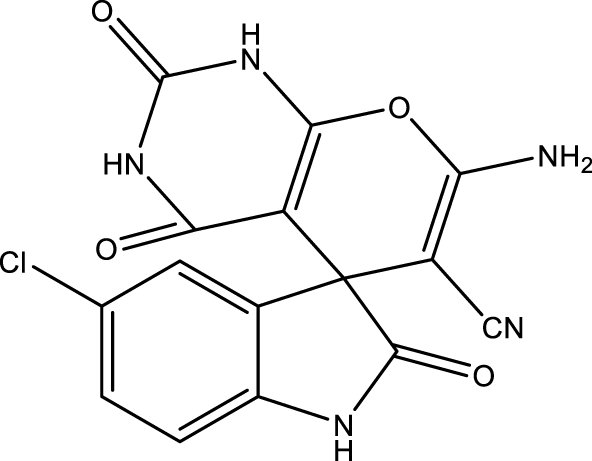	13	94	17.74 × 105	136,460	241–244	242–245 (Dadaei and Naeimi, 2021)
4f	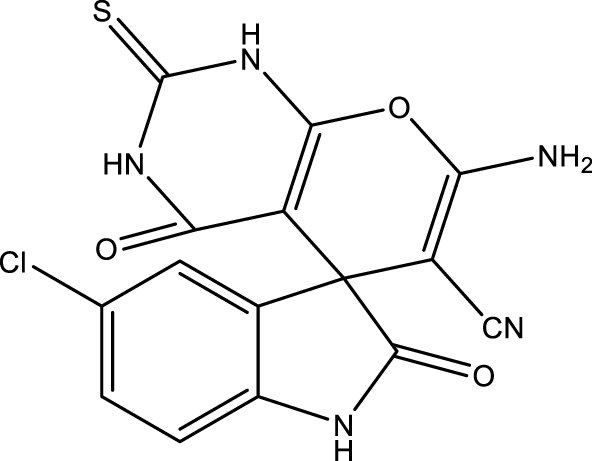	13	93	17.55 × 105	135,000	231–232	228–230 (Dadaei and Naeimi, 2021)
4g	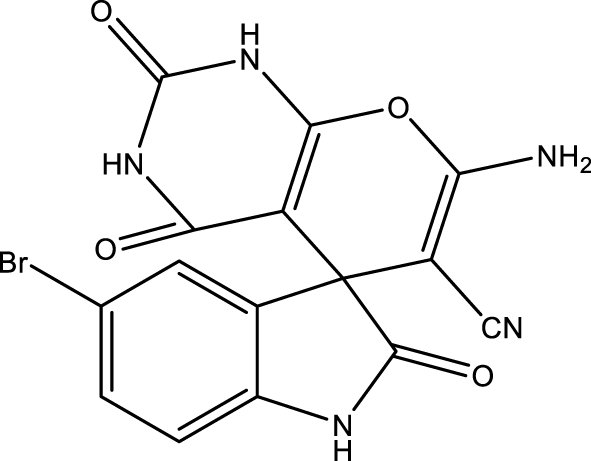	15	90	16.98 × 105	113,200	262–264	263–265 (Bodaghifard and Mousavi, 2020)
4h	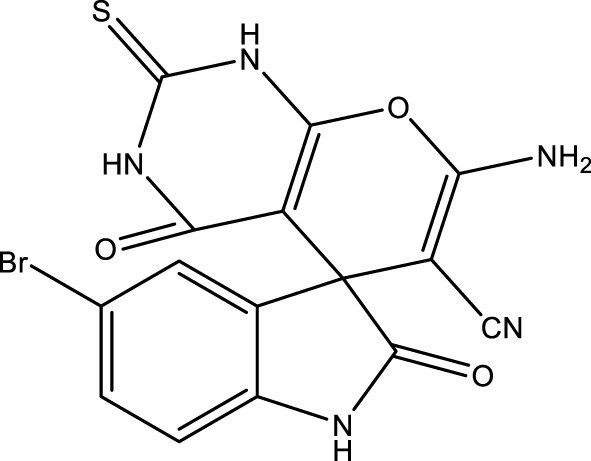	15	92	17.35 × 105	115,600	240–243	243–245 (Bodaghifard and Mousavi, 2020)
4i	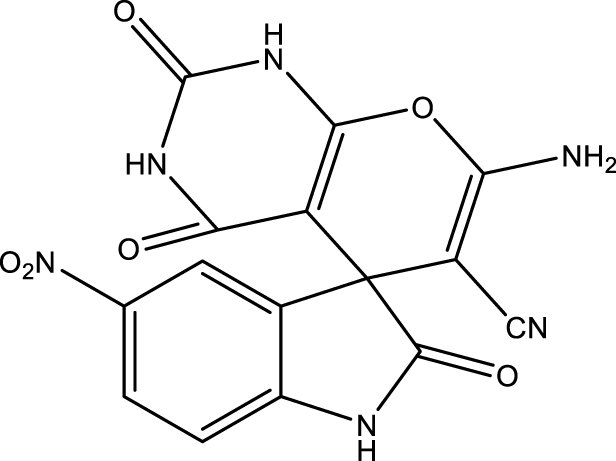	14	94	17.74 × 105	126,700	285–287	288–289 (Keshavarz, 2016)
4j	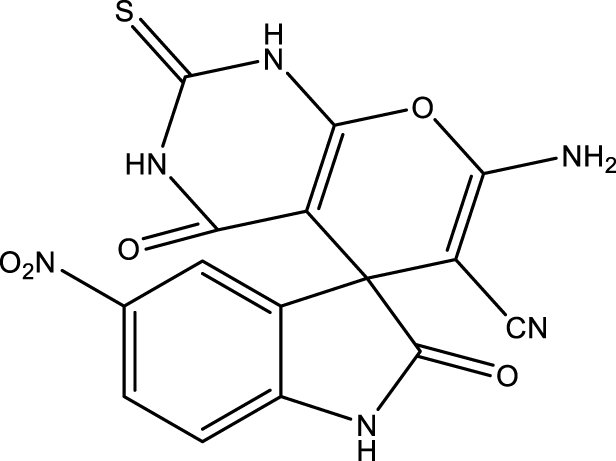	15	95	17.92 × 105	119,460	249–252	252–254 (Azimi and Lasemi, 2022)
4k	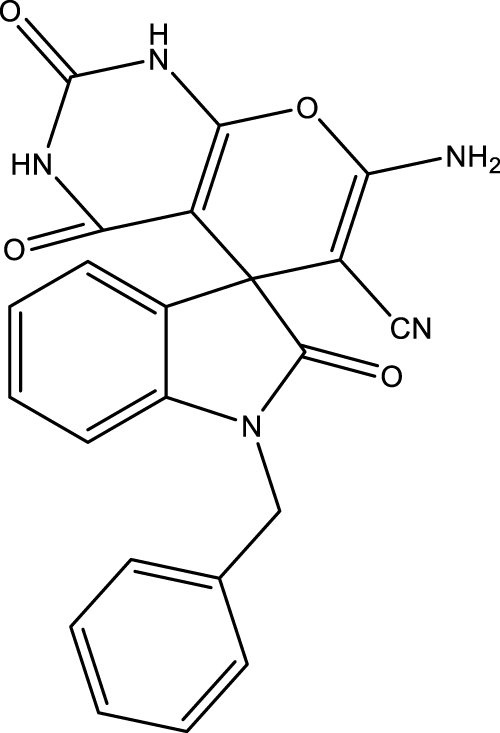	17	89	16.79 × 105	98,760	279–280	In this study
4L	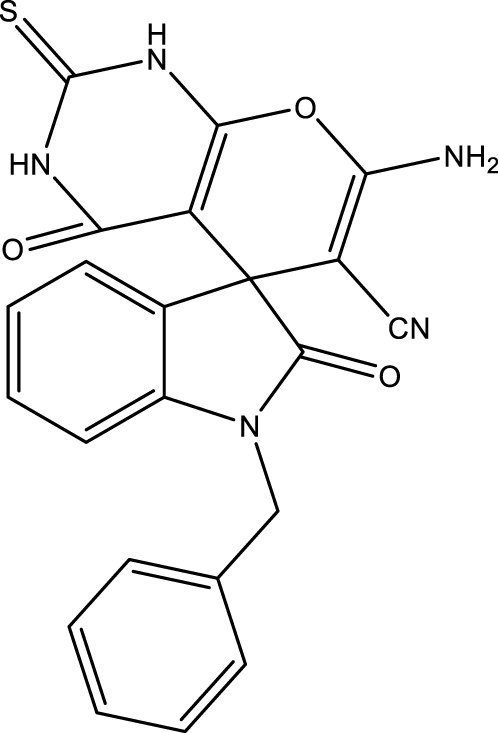	20	85	16.04 × 105	80,200	286–288	In this study

The proposed mechanism for synthesizing spiro [indoline-pyranopyrimidines] derivatives using Fe_3_O_4_/Zn-metal organic framework magnetic nanostructures as a magnetic nano-catalyst was given in Scheme 1.

There have been several reports of the three-component reaction of malononitrile, indoline-2,3-dione derivatives, and barbituric acid or thiobarbituric acid for the synthesis of spiro [indoline-pyranopyrimidines] derivatives*.* Some of them that were reported recently, were listed in Table 4 and compared with this study.

**TABLE 4:** Reported methods for the synthesis of compound 4a.

Entry	Catalyst	Time (min)	Temperature (°C)/Condition	Yield (%)
1	Silica-supported organocatalyst	60	80	98 (Khalafi-Nezhad et al., 2013)
2	Glutathione functionalized Fe_3_O_4_ nanoparticles	15	80	97 (Jamatia et al., 2016)
3	Tin dioxide in ethanol	90	20	96 (Moradi et al., 2019)
4	C3H6N6*3C5H9NO2	10	20	95 (Nagaraju et al., 2017)
5	Pyridine-2,3-dicarboxylic acid	6	70	95 (Oudi et al., 2019)
6	Zinc (II) immobilized on poly (ureaformaldehyde)-functionalized silica-coated CoFe_2_O_4_	30	Reflux (water)	91 (Bodaghifard and Mousavi, 2020)
7	1-Amino-2-naphthol-4-sulfonic acid supported on magnetic nano-CoFe2O4 nanocatalyst	10	40	89 (Faroughi Niya et al., 2021)
8	Fe_3_O_4_/Zn-metal organic framework magnetic nanostructures (this work)	10	r.t	97

The comparison between the results proves that the catalyst of this study has better conditions for the synthesis of derivatives and high efficiency, and less time was its advantages.

**FIGURE 10:**
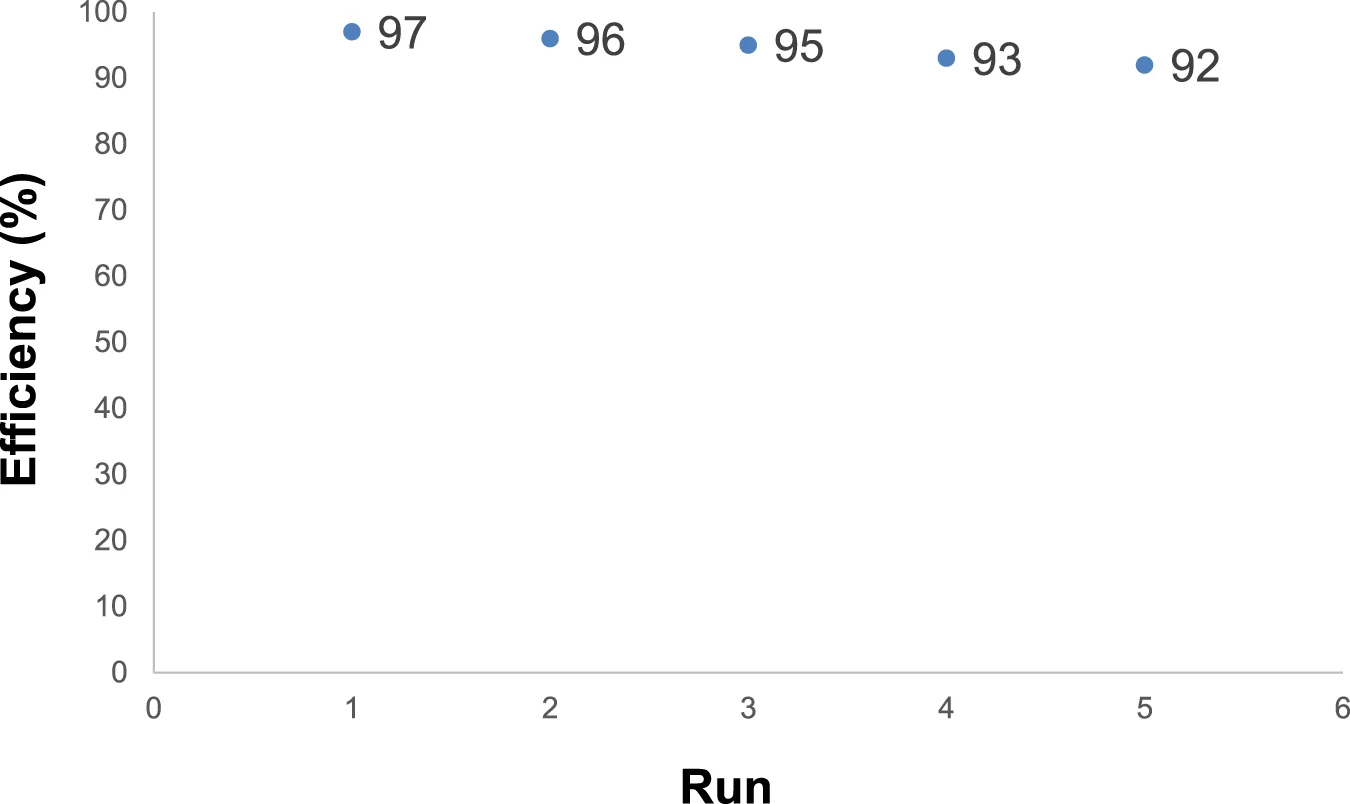
Fe_3_O_4_/Zn-metal organic framework magnetic nanostructures reusability in the synthesis of compound 4a.

Magnetic Fe_3_O_4_/Zn-metal organic framework magnetic nanostructures showed significant recycling properties. The Fe_3_O_4_/Zn-metal organic framework magnetic nanostructures, after acting as a catalyst, were collected by a magnet, and washed several times with water and ethanol then reused in the reaction. The results of Figure 10 showed that Fe_3_O_4_/Zn-metal organic framework magnetic nanostructures could be reused up to 5 times.

A hot filtration test was done based on previous reports, and no enhancement in conversion was noticed in the filtrate (Babaei and Mirjalili, 2020). Characterization data such as SEM, XRD, and VSM from catalyst after recycling was done, and it was confirmed that the structure of the Fe_3_O_4_/Zn-metal organic framework magnetic nanostructures was the same as before recycling (Figure 2).

## 4 Conclusion

In short, in this research, new Fe_3_O_4_/Zn-metal organic framework magnetic nanostructures were synthesized using the microwave method and characterization of their structure. It seems that the microwave irradiation synthesis route has a significant effect on the particle size distribution, and morphology, increased specific surface area, and heat stability of samples. In fact, this efficient route can produce samples in a short time with uniform morphology. The effect of the microwave synthesis routes on the morphology and particle-sized distribution is in compared with previous studies.

The high specific surface area of the synthesized nanoparticles made it have high catalytic properties and novel spiro [indoline-pyranopyrimidines] derivatives were synthesized with higher efficiency and less synthesis time than previously reported methods. In this study, spiro [indoline-pyranopyrimidines] derivatives in the 10–20 min with an efficiency of over 85% were synthesized**.**

Another advantage of the specific surface area of Fe_3_O_4_/Zn-metal organic framework magnetic nanostructures was its high antimicrobial properties. In antimicrobial activity on Gram-positive and Gram-negative bacterial strains, MIC values between 16–128 μg/ml, and for fungal strain, MIC value of 128 μg/ml were observed. The results obtained on antibacterial and antifungal activity proved that, Fe_3_O_4_/Zn-metal organic framework magnetic nanostructures, in some cases, had more effective than commercial drugs.

## Data Availability

The original contributions presented in the study are included in the article/Supplementary Material, further inquiries can be directed to the corresponding author.
