# Correction: Application of novel Fe_3_O_4_/Zn-metal organic framework magnetic nanostructures as an antimicrobial agent and magnetic nanocatalyst in the synthesis of heterocyclic compounds

**DOI:** 10.3389/fchem.2026.1952141

**Published:** 2026-10-05

**Authors:** Bashar S. Bashar, Hawraa A. Kareem, Yaser Mohamad Hasan, Nafis Ahmad, A. M. Alshehri, Kadhum Al-Majdi, Salema K. Hadrawi, Munthir Mohammed Radhy AL Kubaisy, Maytham T. Qasim

**Affiliations:** 1 Department of Computing Technologies Engineering, Al-Nisour University College, Baghdad, Iraq; 2 Anesthesia Techniques Department, Al-Mustaqbal University College, Babylon, Iraq; 3 Technical Engineering College, Al-Farahidi University, Baghdad, Iraq; 4 Department of Physics, College of Science, King Khalid University, Abha, Saudi Arabia; 5 Department of Biomedical Engineering, Ashur University College, Baghdad, Iraq; 6 Refrigeration and Air-conditioning Technical Engineering Department, College of Technical Engineering, The Islamic University, Najaf, Iraq; 7 The University of Mashreq, Baghdad, Iraq; 8 Department of Anesthesia, College of Health and Medical Technology, Al-Ayen University, Thi-Qar, Iraq

**Keywords:** Fe3O4/Zn-metal organic framework magnetic nanostructures, microwave assisted, antimicrobial agent, MIC and MBC value, spiro[indoline-pyranopyrimidines

In section **2 Experimental**, *2.1 General*, paragraph 1, the sentence “Hitachi S-4800 FESEM (Field Emission Scanning Electron Microscope) for SEM image was used” incorrectly identified the SEM instrument. The SEM micrographs presented in Figure 1 (previously published as Figure 6), panels I and III, and Figure 2 (previously published as Figure 10), panel I, were acquired using a TESCAN MIRA3 field-emission scanning electron microscope (FE-SEM).

For completeness and reproducibility, the acquisition conditions of the corrected SEM panels are clarified as follows. Figure 1 (I), corresponding to the Fe3O4/Zn-MOF magnetic nanostructures, was acquired at an accelerating voltage of 15.0 kV, a working distance of 4.91 mm, using an InBeam detector, at a magnification of 200 k×, with a 200 nm scale bar. Figure 1 (III), corresponding to the Fe3O4 nanoparticles, was acquired under the same conditions: an accelerating voltage of 15.0 kV, a working distance of 4.91 mm, using an InBeam detector, at a magnification of 200 k×, with a 200 nm scale bar. Figure 2 (I), corresponding to the recovered Fe_3_O_4_/Zn-MOF magnetic nanostructures after catalytic reuse, was acquired at an accelerating voltage of 15.0 kV, a working distance of 4.91 mm, using an InBeam detector, at a magnification of 100 k×, with a 500 nm scale bar. Information regarding conductive coating was not documented in the retained experimental records and therefore cannot be specified reliably.

The particle-size interpretation is also clarified. The average particle size of approximately 24 nm reported in the original article was determined from the SEM image and corresponding particle-size histogram presented in Figure 1 (I–II) for the Fe_3_O_4_/Zn-MOF magnetic nanostructures. This value is in close agreement with the approximately 25 nm crystallite-size estimate obtained from the XRD analysis. The latter represents a crystallite-size estimate and should not be considered identical to the particle size determined from SEM.

Figure 2 (I) was included to examine the morphology of the recovered Fe_3_O_4_/Zn-MOF catalyst after catalytic reuse and was not used to obtain an independent quantitative particle-size measurement. Moreover, Figure 2 (I) was recorded at a lower magnification (100 k×) than Figure 1 (I and III) (200 k×). Therefore, apparent differences in feature dimensions between Figures 1 and 2 should be interpreted in the context of their different magnifications, scale bars, and experimental purposes and do not represent contradictory particle-size measurements.

The corrected paragraph reads: “The SEM images were obtained using a TESCAN MIRA3 field-emission scanning electron microscope (FE-SEM). The micrographs presented in Figures 1 (I, III) and 2 (I) were acquired at an accelerating voltage of 15.0 kV and a working distance of 4.91 mm using an InBeam detector. Figures 1 (I, III) were recorded at a magnification of 200 k×, whereas Figure 2 (I) was recorded at a magnification of 100 k×. Information regarding conductive coating was not documented in the retained experimental records. The FT-IR spectra were recorded by Nicolet AVATAR 360 FT-IR spectrophotometer. An advanced microwave synthesis lab station (MICROSYNTH, Milestone Co.) was used to synthesize Fe_3_O_4_/Zn-metal organic framework magnetic nanostructures. By Bruker FT-NMR Ultra Shield-spectrometer (300 and 75 MHz), the 1H- and 13C-NMR spectra were recorded. Uncorrected melting points of derivatives by KSP1N melting point meter of Krus's type were determined.”

This correction clarifies the SEM instrument designation, the acquisition conditions of the relevant micrographs, and the interpretation of the particle-size information. These clarifications do not alter the experimental data, results, discussion, or conclusions of the original article.

The original version of this article has been updated.

