# Supplementary material for: Application of novel Fe3O4/Zn-metal organic framework magnetic nanostructures as an antimicrobial agent and magnetic nanocatalyst in the synthesis of heterocyclic compounds
Source: Front Chem. 2022 Oct 10;10:1014731. doi: 10.3389/fchem.2022.1014731 (PMC9589061; doi:10.3389/fchem.2022.1014731)
Supplement: Supplementary file 1 [file DataSheet1.PDF]

Supporting information

Application of Novel Fe_3O_4/Zn-Metal Organic Framework Magnetic Nanostructures as Antimicrobial Agent and Magnetic Nanocatalyst in Synthesis of Heterocyclic Compounds

Bashar S. Bashar[1], Hawraa A. Kareem[2], Nafis Ahmad[3], A. M. Alshehri[3], Kadhum Al-Majdi*[4], Salema K. Hadrawi[5,6], Munthir Mohammed Radhy AL Kubaisy[7], Maytham T. Qasim[8], Yaser Mohamed Hasan[9]

[1]Al-Nisour University College, Baghdad, Iraq

[2]Anesthesia Techniques Department, Al-Mustaqbal University College, Babylon, Iraq

[3]Department of Physics, College of Science, King Khalid University, P.O. Box: 9004, Abha 61413, Kingdom of Saudi Arabia

[4]Department of biomedialc engineering, Ashur University College, Baghdad, Iraq

[5]Refrigeration and Air-conditioning Technical Engineering Department, College of Technical Engineering, The Islamic University, Najaf, Iraq

[6]Computer Engineering Department, Imam Reza University, Mashhad, Iran

[7]The University of Mashreq, Baghdad, Iraq

[8]Department of Anesthesia, College of Health and Medical Technology, Al-Ayen University, Thi-Qar, Iraq

[9]Technical engineering college, Al-farahidi University, Iraq

*** Correspondence:**
Kadhum Al-Majdi
Kadhumalmajdi@gmail.com

7'-amino-1-benzyl-2,4'-dioxo-2'-thioxo-1',2',3',4'-tetrahydrospiro[indoline-3,5'-pyrano[2,3-d]pyrimidine]-6'-carbonitrile (4K)

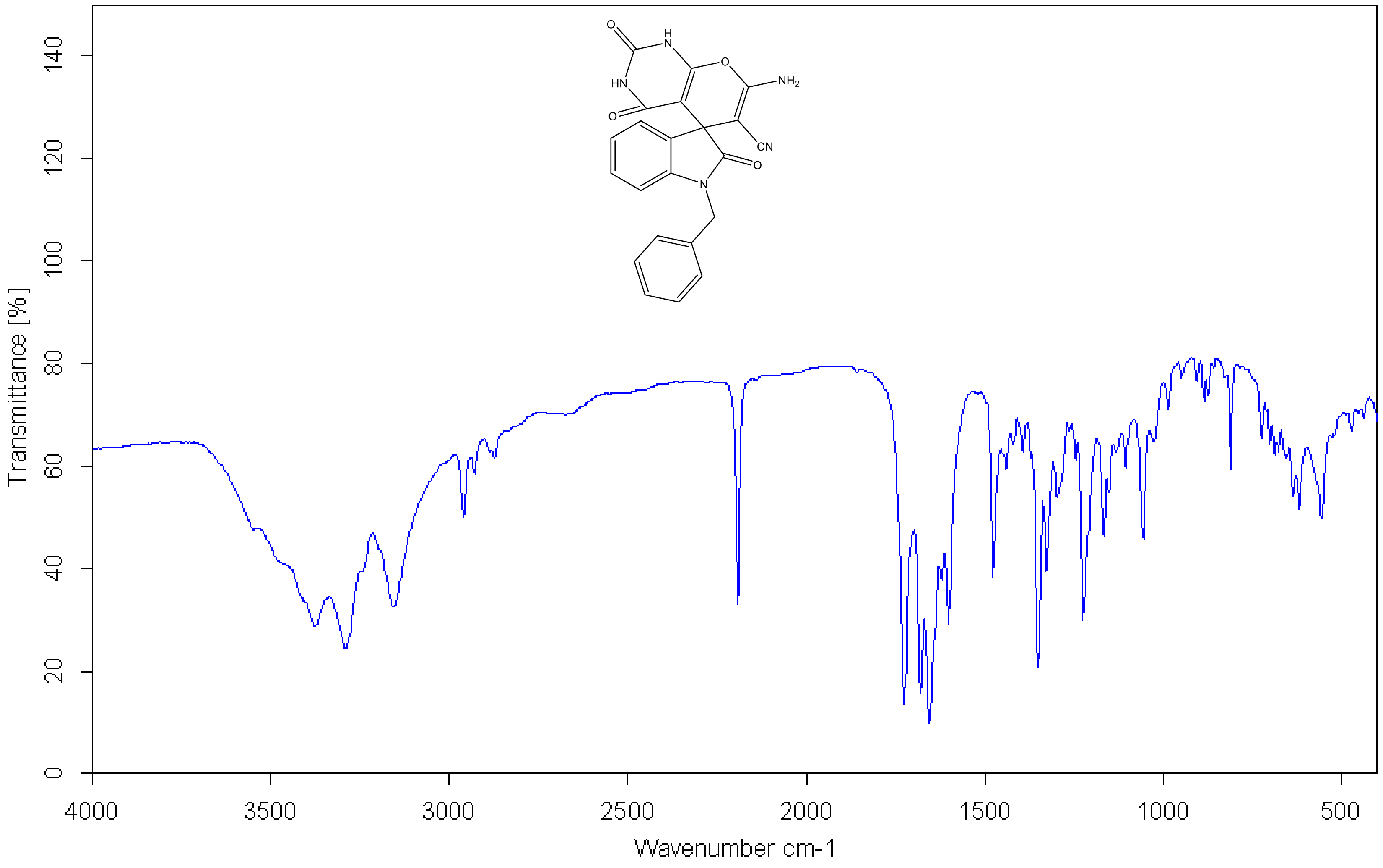

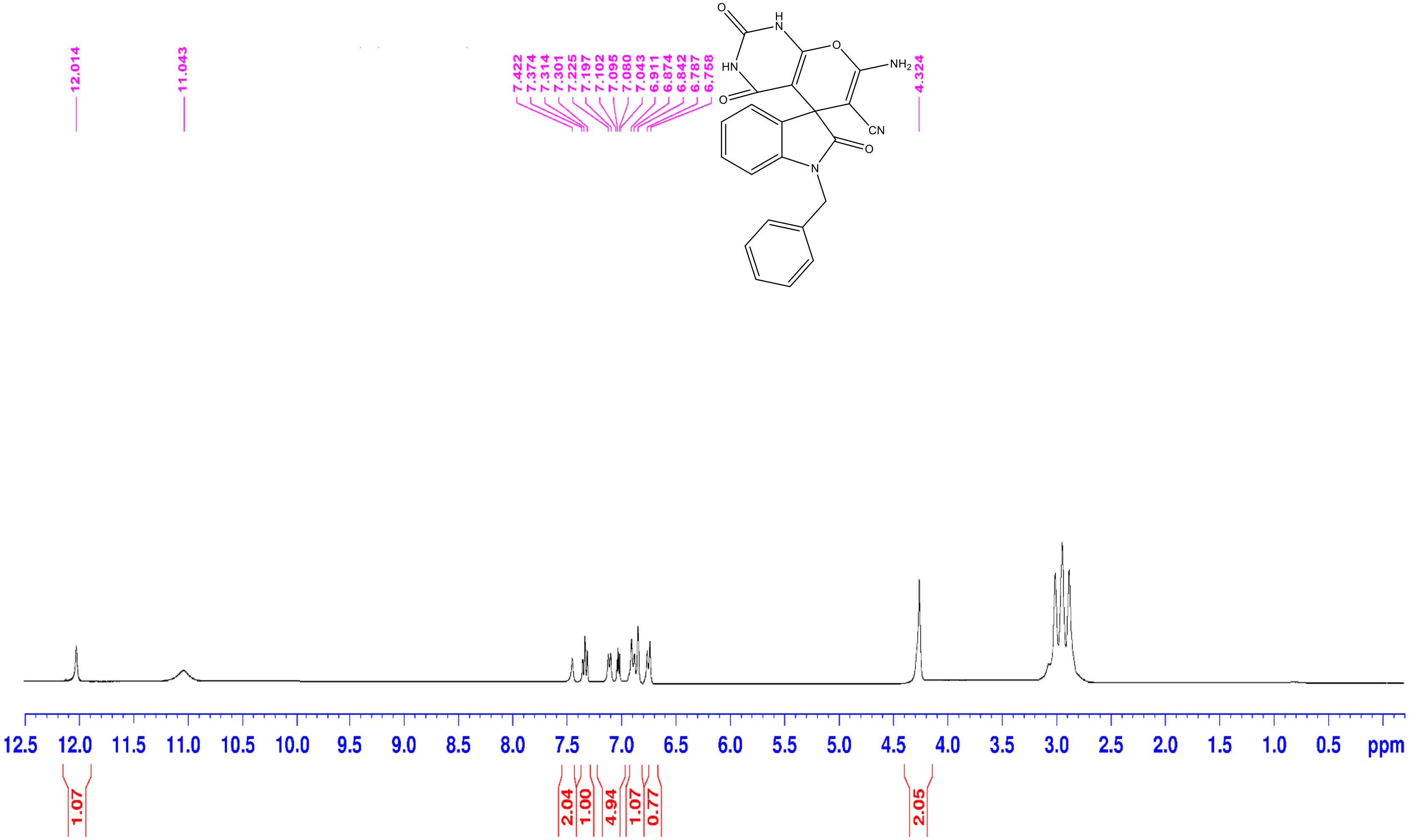

BRUKER

Current Data Parameters

NAME	SB
EXPNO	1529
PROCNO	1

F2 - Acquisition Parameters

Date_	20211030
Time	13.53
INSTRUM	spect
PROBHD	5 mm PABBO BB-
PULPROG	zg30
TD	65536
SOLVENT	DMSO
NS	120
DS	2
SWH	6024.096 Hz
FIDRES	0.091920 Hz
AQ	5.4394879 sec
RG	202
DW	83.000 usec
DE	6.50 usec
TE	295.6 K
D1	1.00000000 sec
TD0	1

======== CHANNEL f1 ========

SFO1	300.8484063 MHz
NUC1	1H
P1	15.00 usec
PLW1	6.40000010 W

F2 - Processing parameters

SI	65536
SF	300.8465480 MHz
WDW	EM
SSB	0
LB	0.30 Hz
GB	0
PC	1.00

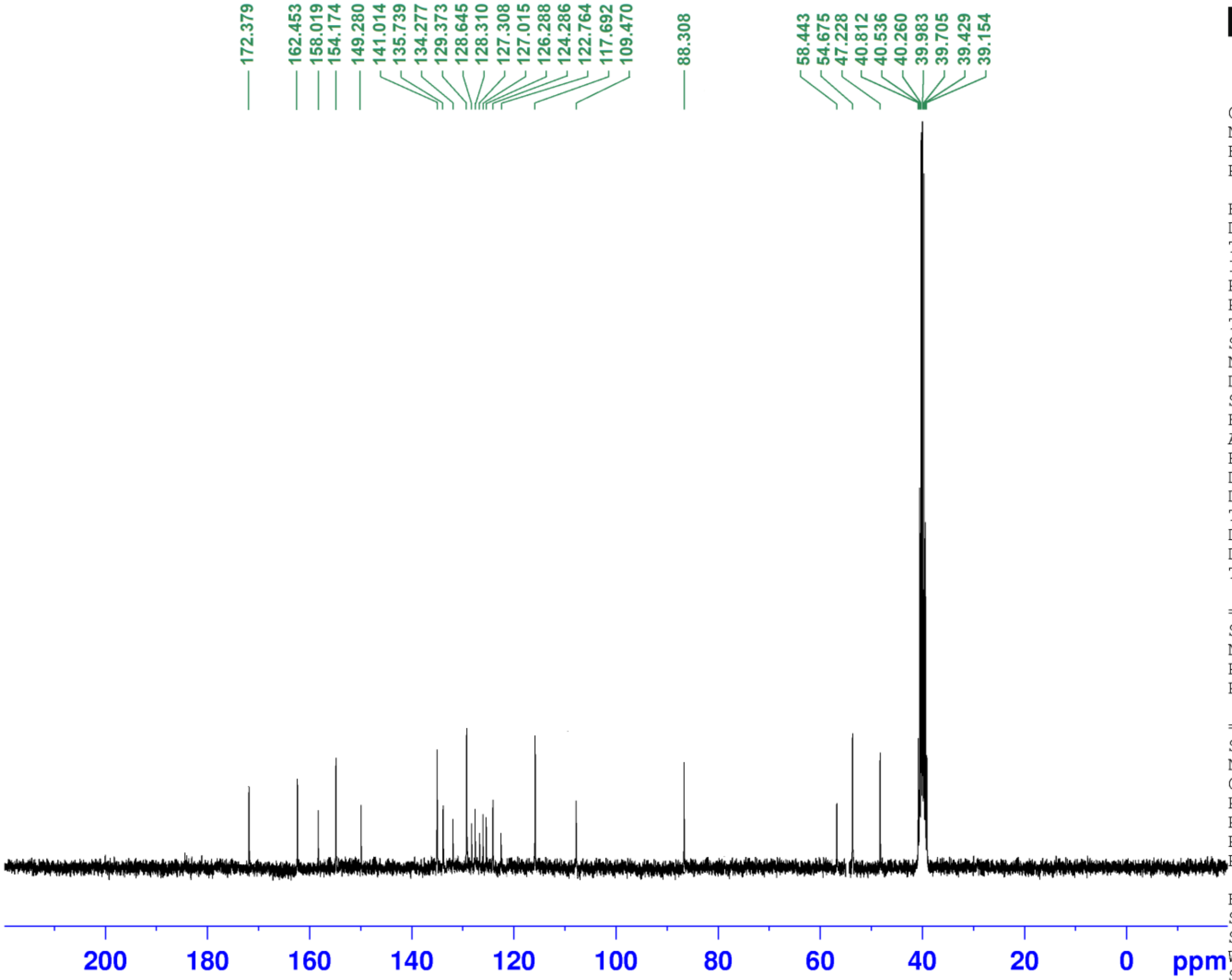


```
Current Data Parameters
NAME                 SB
EXPNO              1531
PROCNO                1

F2 - Acquisition Parameters
Date_          20211030
Time              14.08
INSTRUM           spect
PROBHD    5 mm PABBO BB-
PULPROG          zgpg30
TD                65536
SOLVENT            DMSO
NS                  400
DS                    4
SWH           18115.941 Hz
FIDRES         0.276427 Hz
AQ            1.8087935 sec
RG                  202
DW               27.600 usec
DE                 6.50 usec
TE                296.2 K
D1           2.00000000 sec
D11          0.03000000 sec
TD0                   1

======== CHANNEL f1 ========
SFO1         75.6554892 MHz
NUC1                13C
P1                10.00 usec
PLW1        30.00000000 W

======== CHANNEL f2 ========
SFO2        300.8477518 MHz
NUC2                 1H
CPDPRG[2        waltz16
PCPD2             90.00 usec
PLW2         6.40000010 W
PLW12        0.17778000 W
PLW13        0.14399999 W

F2 - Processing parameters
SI                32768
SF           75.6479250 MHz
WDW                  EM
SSB       0
LB                 1.00 Hz
GB        0
PC                 1.40
```

7'-amino-1-benzyl-2,2',4'-trioxo-1',2',3',4'-tetrahydrospiro[indoline-3,5'-pyrano[2,3-d]pyrimidine]-6'-carbonitrile (4l)

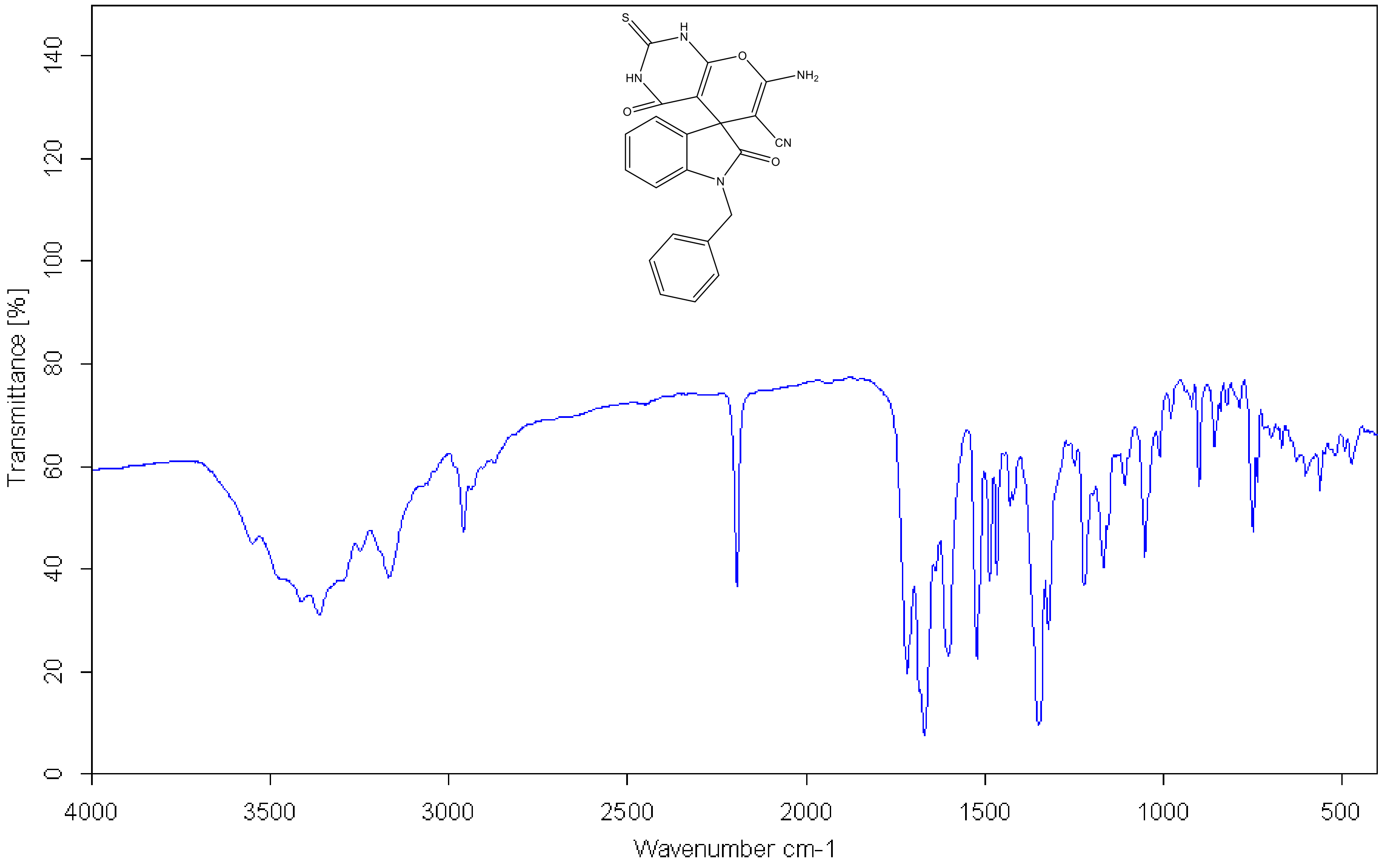

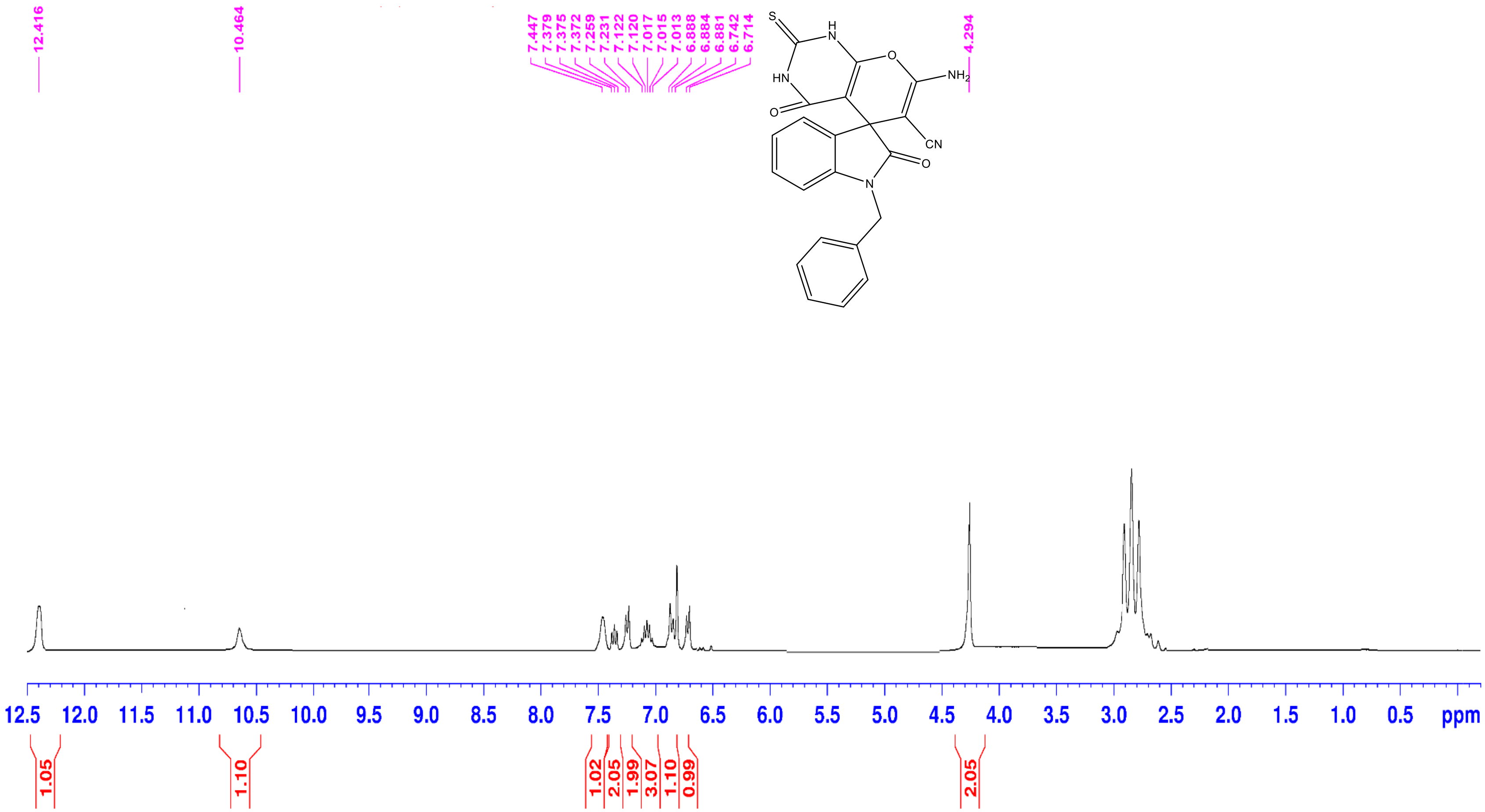

BRUKER

Current Data Parameters
NAME USB
EXPNO 1529
PROCNO 1

F2 - Acquisition Parameters
Date_ 20211030
Time 13.33
INSTRUM spect
PROBHD 5 mm PABBO BB-
PULPROG zg30
TD 65536
SOLVENT DMSO
NS 120
DS 2
SWH 6024.096 Hz
FIDRES 0.091920 Hz
AQ 5.4394879 sec
RG 202
DW 83.000 usec
DE 6.50 usec
TE 295.6 K
D1 1.00000000 sec
TD0 1

======== CHANNEL f1 ========
SFO1 300.8484063 MHz
NUC1 1H
P1 15.00 usec
PLW1 6.40000010 W

F2 - Processing parameters
SI 65536
SF 300.8465480 MHz
WDW EM
SSB 0
LB 0.30 Hz
GB 0
PC 1.00

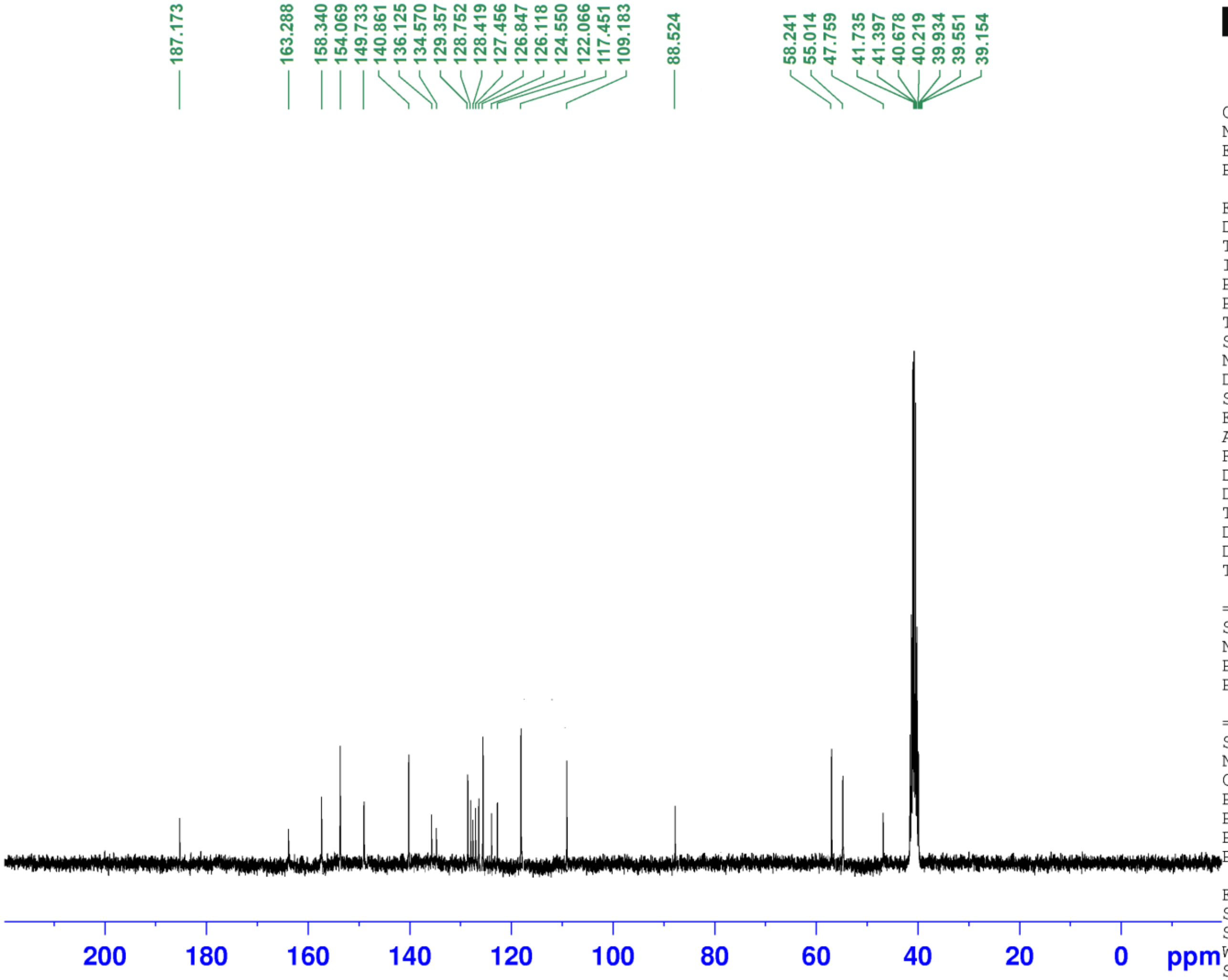

BRUKER

Current Data Parameters
NAME USB
EXPNO 1531
PROCNO 1

F2 - Acquisition Parameters
Date_ 20211030
Time 14.35
INSTRUM spect
PROBHD 5 mm PABBO BB-
PULPROG zgpg30
TD 65536
SOLVENT DMSO
NS 400
DS 4
SWH 18115.941 Hz
FIDRES 0.276427 Hz
AQ 1.8087935 sec
RG 202
DW 27.600 usec
DE 6.50 usec
TE 296.2 K
D1 2.00000000 sec
D11 0.03000000 sec
TD0 1

======== CHANNEL f1 ========
SFO1 75.6554892 MHz
NUC1 13C
P1 10.00 usec
PLW1 30.00000000 W

======== CHANNEL f2 ========
SFO2 300.8477518 MHz
NUC2 1H
CPDPRG[2 waltz16
PCPD2 90.00 usec
PLW2 6.40000010 W
PLW12 0.17778000 W
PLW13 0.14399999 W

F2 - Processing parameters
SI 32768
SF 75.6479250 MHz
WDW EM
SSB 0
LB 1.00 Hz
GB 0
PC 1.40
